# A Survey Detailing Early Onset Colorectal Cancer Patient and Caregiver Experiences in Canada

**DOI:** 10.3390/curroncol31060238

**Published:** 2024-05-31

**Authors:** Rebecca Auer, Claudia Meszaros, Lucresse Fossouo, Lisa Vandermeer, Barry D. Stein

**Affiliations:** 1 Ottawa Hospital Research Institute, Ottawa, ON K1Y 4E9, Canada; rauer@toh.ca (R.A.); lvandermeer@ohri.ca (L.V.); 2 Department of Surgery, University of Ottawa, Ottawa, ON K1H 8L6, Canada; 3 Department of Epidemiology, Biostatistics and Occupational Health, School of Population and Global Health, McGill University, Montreal, QC H3A 1G1, Canada; claudia.meszaros@mail.mcgill.ca; 4 Colorectal Cancer Canada, Montreal, QC H3Z 2P9, Canada; lufossouo@yahoo.ca

**Keywords:** colorectal cancer, early onset colorectal cancer, cancer patients, cancer treatment

## Abstract

The incidence of early onset colorectal cancer (EOCRC) in Canada has increased. To address the growing incidence of EOCRC, Colorectal Cancer Canada (CCC) developed the Never Too Young (N2Y) program to identify gaps in care and evaluate patient and caregiver experiences with CRC. The survey was available online using SurveyMonkey across Canada between 12 December 2022 and 1 May 2023. The patient and caregiver survey consisted of 113 and 94 questions, respectively. A total of 108 EOCRC patients and 20 caregivers completed the survey. Many respondents were unaware of EOCRC (41.6%) and the disease symptoms (45.2%) before diagnosis. Patient age at diagnosis was between 45 and 50 years in 31.7%, and 72.8% of them were diagnosed at stage III or IV. A perception of an initial misdiagnosis was common (67.4%) for EOCRC patients, and 51.2% felt dismissed due to their age. Patients and caregivers reported impacts of EOCRC on their mental health, with 70.9% of patients expressing a need for support with depression and 93.3% of caregivers experiencing a constant fear of recurrence of their loved one’s cancer. Improving the Canadian population’s awareness of EOCRC (e.g., CRC symptoms) is important for ensuring timely diagnoses. Similarly, it is critical to ensure that healthcare providers are aware of the increase in EOCRC cases and the unique needs of these patients. Re-evaluation of the CRC screening age should be undertaken in Canada to determine whether lowering the start age to 45 years will improve outcomes in this demographic.

## 1. Introduction

Colorectal cancer is the 4th most frequently diagnosed cancers in Canada and the second leading cause of cancer death in men and the 3rd leading cause of death in women. [1]. In 2024, it is projected to impact an additional 24,200 Canadians, with a five-year survival rate of 66% for males and 67% for females and it is estimated that about 9400 Canadians will die form the disease. For Canadians between the ages of 30–49 years colorectal cancer was one of the most commonly diagnosed cancers (9%) [2]. While the prevalence of colorectal cancer in Canadians aged over 50 continues to decline due to the implementation of screening programs, a consistent increase in cases among those under 50 has emerged, leading to the designation of these cases as early onset colorectal cancer (EOCRC). The incidence of EOCRC has been increasing steadily in recent years [3,4,5,6]. A study by Brenner and colleagues in 2017 noticed a increase in colorectal cancer among Canadians born after 1970 [3], with a annual growth rate of 4.45% in women and 3.47% in men [4]. This rise in incidence of EOCRC underscores the need for continuous research and data collection to address this growing public health concern.

People with EOCRC face unique challenges and diagnostic barriers. Many of them, especially those under 50, are unaware of the signs and symptoms of colorectal cancer. This leads to delays in seeking medical help, and as a result, late-stage diagnoses. The American Cancer Society (ACS) recommends screening for colorectal cancer at the age of 45 [7], but in Canada, routine screening for average risk individuals starts at 50 [8]. Detecting EOCRC early can make a big difference in one’s prognosis. In the United States, the Colorectal Cancer Alliance found that over three-fourths of EOCRC patients are diagnosed at stage III or IV of the cancer’s development [9]. If caught early, the survival rate is 92% in early stages but drops to 68% in stage III and a low 11% in stage IV [10]. This highlights the critical importance of early detection and diagnosis in improving patient outcomes.

The medical community’s limited sensitization to EOCRC further compounds the issue, leading to people with CRC being misdiagnosed with other gastrointestinal diseases, exacerbating delays in cancer diagnoses [5]. Colorectal Cancer Canada launched the Never Too Young program in 2018 to increase awareness about EOCRC in Canada, particularly among those under 50. It aims to improve support for patients and help healthcare professionals understand the challenges faced by EOCRC patients [11]. However, despite these efforts, a noticeable gap remains in understanding the experiences of EOCRC patients. This study sought to determine the gaps in clinical and supportive care for Canadian EOCRC patients and review factors that may influence their quality of life.

## 2. Materials and Methods

### 2.1. Survey Design and Questionnaire

The Colorectal Cancer Canada 2022 Never Too Young survey comprised of 201 questions to comprehensively capture a range of information, which delved into respondent demographics, lifestyle, and their journey, encompassing experiences from symptom onset to diagnosis, through treatment, and into post-treatment survivorship. The survey was tailored into two versions: one for completion by current patients or survivors of colorectal cancer, with 113 questions, and a second version containing 94 questions, of which 77 questions were designated for caregivers responding on behalf of the patient and 17 questions tailored to the caregivers’ unique perspective. Respondents were given the option to complete the survey in either French or English for enhanced accessibility.

### 2.2. Study Population and Participant Recruitment

The focus of this study was on young adults in Canada, particularly those aged 18–50, who were diagnosed EOCRC. Invitations to participate were extended to individuals residing in Canada, capable of providing virtual consent. The inclusion criteria for caregivers mirrored those of the EOCRC patients, requiring caregivers to be caretakers of patients aged 18–50, able to provide virtual consent, and living in Canada. Participants were recruited through various channels facilitated by Colorectal Cancer Canada. These channels encompassed social media, newsletters, live events, interaction with Colorectal Cancer Canada’s employees and ambassadors, and the involvement of members on Colorectal Cancer Canada’s advisory panel.

### 2.3. Data Collection

The survey was disseminated between 12 December 2022 and 1 May 2023 using SurveyMonkey, with accessibility extending to all provinces and territories. Participants could access the survey through a survey link or QR code. Demographic information collected for each respondent included family history of colorectal cancer, current age, race/ethnicity, gender, marital status at diagnosis, parenthood status, educational attainment, province of residence, community type (urban, rural, or suburban), and current employment status. Furthermore, the survey delved into diagnostic journeys, treatment experiences, and assessed various lifestyle factors including as physical activity and smoking and alcohol consumption. The survey form is included under the Supplementary Material S1.

### 2.4. Data Analysis

**Figure 1:**
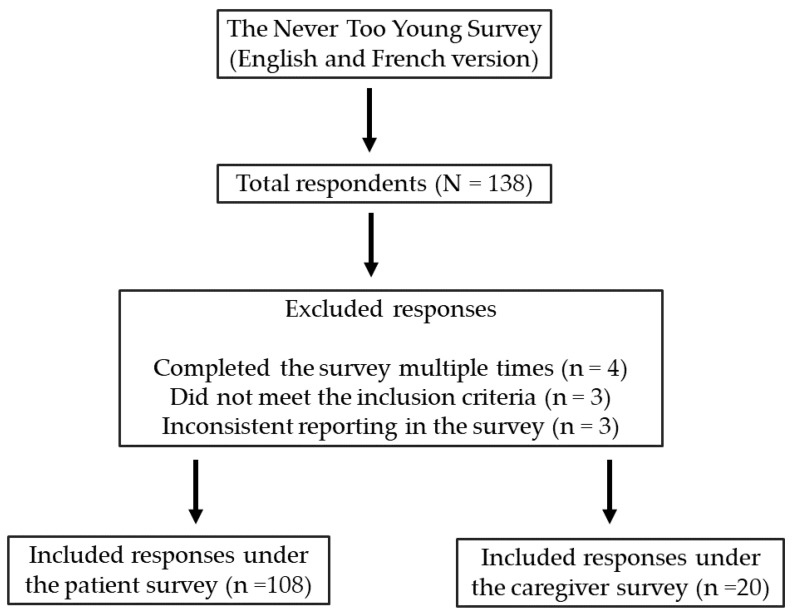
Flowchart of survey respondents included in the study.

A total of 138 responses were collected; after careful review, 10 responses were omitted from the analysis. Of these exclusions, four were related to individuals who had submitted multiple responses, with only their initial responses included. Additionally, three respondents were removed for not meeting the age criteria (diagnosed after age 50), and three more were excluded due to inconsistent survey responses. The final dataset included 128 eligible respondents for analysis. All patient responses for all survey versions were exported from SurveyMonkey as an Excel file. A descriptive analysis was conducted using Excel, and all figures were generated using Canva^®^. Figure 1 shows a flowchart of survey respondents included in the study.

### 2.5. Ethics Approval

Ethics approval was obtained from the Ontario Cancer Research Ethics Board on 31 August 2022. The Clinical Trials Ontario (CTO) project ID number is 4108.

## 3. Results

### 3.1. Respondent Demographics

**Figure 2:**
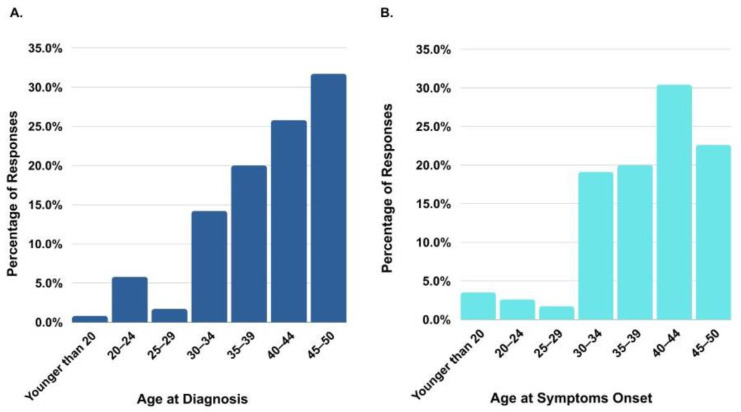
Age distribution for symptoms onset and diagnosis. (**A**) Age at diagnosis (N = 120). (**B**) Age at symptoms onset (N = 115).

While majority of the respondents were white (87.4%) females (63.5%) from Ontario, the survey was disseminated across Canada with respondents from 9 other provinces, including Quebec, Alberta, British Columbia, Saskatchewan, Manitoba, and the Maritimes. Of the respondents, 84.5% were patients (with 53.9% undergoing active cancer treatment and 30.5% in survivorship), while 15.6% were caregivers. Of the female respondents, a portion were married (73.4%) with children (69.0%) at the time of their CRC diagnosis. Over half of them held university degrees (53.6%). An array of initial symptoms was reported by respondents, with the top three most common symptoms being blood in stool (18.9%), bloating and/or gas (11.9%), and weakness and/or fatigue (11.6%) (Supplementary Figure S1). Approximately 25% were diagnosed between 40 and 45, and over 30% were diagnosed between 45 and 50. The mean age at symptom onset was 38.8 years, with 30.4% of respondents first noticing symptoms between the ages of 40–44 (Figure 2A,B).

### 3.2. EOCRC Awareness

Among the respondents, 41.6% were unaware that colorectal cancer could occur in individuals under 50, and 45.2% were unfamiliar with the signs and symptoms of CRC before their diagnosis. Additionally, 72.9% of patients did not discuss their family health history, including colorectal cancer risk factors, with family, friends, or doctors prior to their diagnosis. Post-diagnosis, only 9.3% of patients reported discussing their family health history and colorectal cancer risk factors with their doctors (Supplementary Table S2).

### 3.3. Diagnosis and Patient Experiences towards Receiving a Diagnosis

Approximately 57% of respondents received a diagnosis of colon cancer, while 40.3% were diagnosed with rectal cancer. Notably, a substantial 72.8% of the diagnoses occurred at later stages, with 48.0% at stage III and 24.8% at stage IV (Supplementary Table S3).

Upon experiencing symptoms, 56.2% sought consultation with a primary care provider, but 33.7% waited for more than 6 months before seeking medical attention. Over two-thirds (70.7%) had consultations with at least 2 doctors before eventually receiving a CRC diagnosis. In 16.7% of cases, patients attended 3 or more appointments before their doctor suspected CRC, while 9.8% endured more than 10 appointments. In 31.1% of cases, it took over 6 months to obtain a colorectal cancer diagnosis after seeking medical care. Notably, a rectal exam was not performed for 52.9%, and 45.2% reported feeling that their doctor did not appear concerned after describing their signs and symptoms. Over half (51.2%) believed that their doctors dismissed signs and symptoms of colorectal cancer solely due to patient age. Furthermore, 67.4% felt that they received an incorrect diagnosis, with 22.1% reporting a misdiagnosis of hemorrhoids. Detailed information on the path to diagnosis for EOCRC patients is presented in Table 1.

### 3.4. Health History and Behavioral Risk Factors for Colorectal Cancer

A family history of colorectal cancer in first and second degree relative was noted in 41.9% of respondents. In terms of physical activity, 36.3% were lightly active, while 33.9% were moderately active. Most respondents were non-smokers at the time of their diagnosis (89.7%), with 67.6% having never smoked. The proportion of those who consumed alcohol (54.5%) and those who did not (45.5%) was similar. Approximately 75.4% of respondents did not adhere to a specific diet before their diagnosis (Supplementary Table S2).

### 3.5. Treatment Experiences of Early Age Onset Colorectal Cancer Patients

A majority reported understanding and feeling fully informed about their treatment plan (75.2%), and 84.3% did not seek a second opinion during the treatment process. Challenges associated with treatment included anal leakage of stools (47.4%), sore skin around the anal area (66.7%), frequent nighttime bowel movements (72.5%), and feelings of embarrassment due to bowel movements (78.9%). Challenges with stoma bags and skin conditions were prevalent, with most respondents encountering issues like leakage of stools from the stoma bag (77.8%), sore skin around the stoma (80%), and challenges in caring for the stoma (56.8%). The majority reported an impact on daily life, affecting social activities (61.1%), recreational and sports activities (65.7%), and personal relationships (57.1%) (Supplementary Table S4).

### 3.6. Sexuality and Fertility Experiences of Early Age Onset Colorectal Cancer Patients

The discussion with healthcare providers about sexual side effects prior to treatment revealed variations, with 49% of colon cancer and 20% of rectal cancer patients reporting providers did not discuss any potential sexual side effects of treatment. Of those having discussed potential sexual side effects of treatment, 9% of colon cancer and 19% of rectal cancer patients were informed of potential loss of sexual function, and 16% of colon cancer and 28% of rectal cancer patients were informed of potential infertility. Post-treatment experiences indicated that 13% of colon cancer and 22% of rectal cancer patients experienced painful sex; these respondents were mostly female (92.3%). In total, 49% of colon cancer and 33% of rectal cancer patients reported a decrease in sex drive, and 13% of rectal cancer patients indicated a total loss of sexual function. Fear of participating in sexual activities after guidance from medical staff remained present for 53% of rectal cancer patients. Regarding relationships, 58% of colon cancer and 41% of rectal cancer patients never felt a strain due to sexual dysfunction. However, 39% of colon cancer and 70% of rectal cancer patients who responded felt their sexual dysfunction affected their ability to become intimate. Discussion on fertility preservation with medical professionals was reported by 32% of colon cancer and 36% of rectal cancer patients. Treatment-induced sterility or infertility was reported by 10% of colon cancer and 52% of rectal cancer patients (Table 2).

### 3.7. Patient Mental Health Experiences

The mental health experiences of patients revealed significant challenges. All the patients reported experiencing emotional exhaustion, with 36.0% stating they always felt exhausted, and 90.1% reported feeling withdrawn from others. Many expressed a need for support with depression (70.9%). Concerns about cancer recurrence are notable, with 93.6% fearing its return, and this occasionally interferes with daily activities (65.5%). Sudden feelings of panic and anxiety are common (82.9%) (Supplementary Table S5).

### 3.8. Caregiver Mental Health Experiences

Most caregivers (87.5%) were concerned about their loved one’s mental health. Emotionally, 31.3% of caregivers always felt exhausted. Depression affected 37.5% of caregivers. The fear of cancer recurrence was high, with 93.3% always having this fear. Fatigue impacted social activities for 43.8% of caregivers (Supplementary Table S6).

### 3.9. Financial Health

At the time of diagnosis, 85% of patients were employed, and 73.3% of caregivers were employed. A significant portion of patients (81.6%) had to take a leave of absence, quit their job, or leave school due to the diagnosis. Notably, 33.0% of patients found the Canada Employment Sickness Benefit insufficient. The majority (57.1%) reported that their employer or school helped accommodate their treatment schedule. Financial stress was common, with nearly 40% expressing concerns about their financial stability, and 36.5% were concerned about the adequacy of public insurance (Supplementary Table S7).

## 4. Discussion

This survey deepens our understanding of the challenges faced by patients with EOCRC and their caregivers, building upon the 2020 Never Too Young (N2Y) survey by Colorectal Cancer Canada. Our findings highlight significant gaps in care and support, emphasizing the urgency of a more patient-centered, integrated approach. Key findings include, low awareness of EOCRC risk, prolonged diagnostic journeys, physical and emotional treatment challenges, substantial mental health concerns, and financial burden among patients and caregivers [11]. These results underscore the pressing need for a comprehensive, patient-centered approach to EOCRC care.

A significant issue highlighted by the survey is the delayed diagnosis of EOCRC, as over 40% of individuals were uninformed about the possibility of CRC occurring before the age of 50, and they also exhibited unfamiliarity with its symptomatic manifestation. This observation is consistent with previous findings reported by the Colorectal Cancer Alliance in the United States, demonstrating that 49% of individuals lacked awareness of the signs and symptoms of CRC [9]. Moreover, an earlier survey conducted by CCC found that 32.9% of individuals were unaware of the potential for CRC to affect individuals under the age of 50 [6]. These comparisons highlight a persistent issue of insufficient awareness and knowledge regarding EOCRC, underscoring the need for targeted educational and awareness initiatives.

The data also reflect delays in seeking care, with nearly 29% waiting over six months before seeking medical attention and enduring multiple visits before receiving a CRC diagnosis. This observation aligns with the previous N2Y report that showed 28.8% of individuals waited more than six months before seeking medical attention [6]. This delay may be attributed to the generic nature of the colorectal cancer symptoms, such as diarrhea, weakness, bloating, abdominal cramps, and blood in the stool, which can overlap with various gastrointestinal diseases [12,13]. The absence of rectal examinations combined with perceived dismissal by healthcare practitioners, raises concerns about the risk of misdiagnosis.

Furthermore, EOCRC patients face unique challenges due to their younger age, in line with earlier reports that underscore their frequent dismissal by healthcare providers and misdiagnosis with other conditions, such as hemorrhoids [6,9,14]. Earlier reports showed an increase in CRC incidence in adults aged 20–49 years in Europe, the largest increase among 20–39 year old [15]. Several studies have reported that younger patients present more often with stage III or IV disease, consistent with the present data [16,17,18]. Given the young age at which patients experience symptoms and the sporadic nature of the disease, the recommended screening age of 50 in Canada may be inadequate to capture the growing population of EOCRC individuals. These data also highlight the necessity of sensitizing the healthcare community to the increasing incidence of EOCRC and the need for further research to ascertain whether lowering the age for average risk individuals, as recommended in the United States at 45 years of age, would enhance early diagnosis [7].

Beyond diagnostic challenges, the survey highlights the substantial mental health burden experienced by EOCRC patients and their caregivers, including emotional exhaustion, withdrawal, and depression. The pervasive fear of cancer recurrence and sudden episodes of panic and anxiety highlight the acute need for integrated psychological support [19,20,21,22]. Patients often grapple with anxiety, depression, fear of recurrence, and emotional exhaustion following their cancer diagnosis. A survey by the Colorectal Cancer Alliance in the United States has shown that 75% of EOCRC patients were worried about their psychological health, 64% required assistance for depression, and 74.4% experienced abrupt episodes of panic and anxiety [9]. The findings underscore that patients require better mental health support, psychosocial assistance, and financial guidance, with many expressing concerns about these inadequacies [23,24]. Moreover, the lasting side effects of treatment and changes in sexuality can further exacerbate the poor mental health of cancer patients and survivors [25,26,27]. The survey data highlight the pressing necessity for effective communication about sexual side effects and fertility preservation in the context of cancer treatment. Notably, our results reveal a substantial gap in these discussions, with 42% of colon cancer and 19% of rectal cancer patients reporting no communication on sexual side effects by healthcare providers. This emphasizes the critical need for enhanced patient-provider communication and effective communication about sexual side effects and fertility preservation in the context of cancer treatment.

The financial implications of an EOCRC diagnosis are substantial, contributing to what is sometimes referred to as financial toxicity. Patients frequently experience a heightened financial burden, often resulting in unemployment and diminished income due to their cancer diagnosis [28,29]. This survey aligns with prior reports, emphasizing the overarching theme of financial toxicity in this demographic as 48.2% of patients and caregivers that responded indicated that they felt financially stressed and 55.0% worry about the future of their finances. A considerable percentage found existing financial support systems inadequate, exacerbating their burden. Addressing these gaps with evidence-based interventions is essential to provide EOCRC patients and their caregivers with comprehensive support throughout their cancer journey.

The study enriches our understanding about the challenges faced by EOCRC patients and their caregivers. It takes a comprehensive approach, exploring various dimensions of their journey, identifying critical gaps in care, and paving the way for targeting interventions to address these challenges effectively. A notable strength lies in its inclusion of the caregiver perspective. Caregivers play a pivotal role in a patient’s journey, yet their experiences and needs are often overlooked. By incorporating their viewpoint, this study offers a more comprehensive understanding of their challenges. Nonetheless, the study is not without limitations. As majority of the respondents were white (87.4 %) females (63.5 %) from Ontario, there is selection bias that may constrain the generalizability of the findings to the wider Canadian population, which may lead to different results. Additionally, a heavy reliance on self-reported data introduces the possibility of recall bias. Individuals who chose not to participate in the survey due to their health deteriorating or those who passed away during their cancer journey might hold distinct experiences and perspectives that could introduce non-response bias.

To address these identified gaps and build upon the strengths of this study, we propose a set of recommendations. These recommendations serve as a practical guide for healthcare providers and support organizations in improving the quality of care and support for EOCRC patients and their caregivers. The following table outlines the gaps and corresponding recommendations (Table 3).

## 5. Conclusions

In summary, this survey offers a holistic view of the multifaceted challenges faced by EOCRC patients and caregivers. Patient-centered approaches are needed to comprehensively address these complex challenges. This approach should cover early detection, tailored treatment, better communication between patients and providers, robust mental health support, and financial assistance, forming a safety net that eases the physical, emotional, and financial burden experienced by EOCRC patients and their caregivers during their cancer journey.

## Figures and Tables

**Table 1:** Path to diagnosis of early age onset colorectal cancer patients.

Variable.	Total N	%
Doctor seen at symptom onset		
Primary care provider	93	56.2
Pediatrician	1	0.6
Gastroenterologist	16	9.9
Emergency room doctor	28	17.3
OBGYN	3	1.9
Urologist	1	0.6
Urgent care	9	5.6
Surgeon	6	3.7
Other	7	4.3
Time between symptoms onset and seeking medical attention		
<1 month	22	21.8
1–3 months	30	29.7
3–6 months	15	14.9
6+ months	34	33.7
Received a rectal exam prior to diagnosis		
Yes	45	43.3
No	55	52.9
N/A	4	3.8
Doctor that led to colonoscopy		
Family/primary care provider	54	45.0
Gastroenterologist	21	17.5
Emergency room doctor	16	13.3
OBGYN	1	0.8
Urgent care with ER	1	0.8
Pediatrician	1	0.8
Surgeon	21	17.5
Other (please specify)	5	4.2
Level of concern by doctors following description of symptoms		
They did not seem concerned	47	45.2
They seemed slightly concerned	22	21.2
They seemed moderately concerned	23	22.1
They seemed very concerned	12	11.5
Felt that doctors dismissed the signs and symptoms of colorectal cancer due to patient age		
Yes	63	51.2
No	52	42.3
I do not know	7	5.7
Yes, but for a reason other than age	1	0.8
Mistaken diagnosis		
None of the below	56	32.6
Hemorrhoids	38	22.1
Symptoms of childbirth	8	4.7
Appendicitis	3	1.7
Gynecological issues	7	4.1
IBS	10	5.8
IBD	3	1.7
Mental health issues	7	4.1
Crohn’s/Colitis	11	6.4
Other	29	16.9

**Table 2:** Impacts of treatment on sexuality, fertility, and experience towards the given guidance.

Variable	Colon Cancer		Rectal Cancer	
	Total N	%	Total N	%
Did any of your providers discuss sexual side effects due to radiation and surgery before treatment?				
Painful sex	1	1%	>10	15%
Decrease in sex drive	3–10	7%	>10	17%
Loss of sexual function	3–10	9%	>10	19%
Infertility	>10	16%	>10	28%
I do not remember	>10	16%	1	1%
Providers did not discuss any of the above	>10	49%	>10	20%
Did you experience any of the following after treatment?				
Painful sex	3–10	13%	>10	22%
Decrease in sex drive	>10	49%	>10	33%
Loss of sexual function	3–10	13%	>10	20%
Infertility	2	3%	>10	17%
I do not remember	3–10	16%	3	3%
Prefer not to answer	3–10	6%	3–10	4%
Sexual function issues after treatment and surgery				
No loss of sexual function	32	56%	16	35%
Partial loss of sexual function	15	26%	16	35%
Total loss of sexual function	0	0%	6	13%
Prefer not to answer	3	5%	4	9%
Even with guidance from medical staff, I am still afraid to have sex				
Always	0	0%	4	10%
Often	3	7%	8	21%
Sometimes	4	9%	9	23%
Never	33	73%	15	38%
Prefer not to say	5	11%	3	8%
Sexual dysfunction puts a strain on relationships				
Always	2	5%	4	10%
Often	2	5%	7	18%
Sometimes	11	28%	11	28%
Never	23	58%	16	41%
Prefer not to say	2	5%	1	3%
Sexual dysfunction affects ability to become intimate with others				
Always	1	2%	6	16%
Often	3	7%	9	24%
Sometimes	12	29%	11	30%
Never	22	54%	10	27%
Prefer not to say	3	7%	1	3%
Feel awkward to receive guidance for sexual life				
Always	2	5%	4	12%
Often	3	7%	2	6%
Sometimes	12	28%	11	33%
Never	22	51%	15	45%
Prefer not to say	4	9%	1	3%
I believe that I am not a complete person due to sexual dysfunction				
Always	1	3%	7	18%
Often	3	8%	4	11%
Sometimes	4	10%	11	29%
Never	29	73%	15	39%
Prefer not to say	3	8%	1	3%
I worry that I am not enough for a significant other due to sexual dysfunction				
Always	2	5%	7	18%
Often	2	5%	10	26%
Sometimes	10	25%	8	21%
Never	21	53%	12	32%
Prefer not to say	5	13%	1	3%
Medical professional discussed fertility preservation during diagnosis or treatment				
No	27	43%	22	47%
Reproductive health issues prior to treatment				
No	39	62%	33	69%
Treatment resulted in sterility or infertility				
No	16	26%	7	15%

**Table 3:** Recommendations for healthcare providers and organizations to improve care for EOCRC patients and their caregivers.

Gaps	Recommendations
Delayed diagnosis	Raise awareness about early onset colorectal cancer (EOCRC) and its symptoms among the general population, targeting individuals under 50. Develop and implement public health campaigns and educational programs.
Misdiagnosis and Dismissal	Sensitize healthcare providers to the increased incidence of EOCRC. Promote comprehensive diagnostic practices, including considering colorectal cancer in younger patients with gastrointestinal symptoms by asking patients about CRC family history.
Lack of Mental Health Support	Integrate mental health support into EOCRC patient care. Provide resources and guidance for managing anxiety, depression, and the fear of recurrence.
Financial Toxicity	Establish financial support systems to mitigate the economic burden on EOCRC patients, such as job loss and reduced income.
Inadequate Communication	Improve patient-provider communication regarding sexual side effects and fertility preservation. Train healthcare professionals to address these sensitive topics with empathy and clarity.
Caregiver Support	Recognize the importance of caregivers in the cancer patient journey. Provide them with support, resources, and mental health assistance to help alleviate their emotional and psychological challenges.

## Data Availability

The data used in this study are not publicly available but may be available upon request. Please contact the corresponding author.

## Supplementary Materials

The following supporting information can be downloaded at: https://www.mdpi.com/article/10.3390/curroncol31060238/s1, Supplementary Material S1: Survey form; Figure S1: Symptoms experienced by early age onset colorectal (EOCRC) patients; Table S1: Respondent demographics for the Never Too Young survey; Table S2: The awareness of early age onset colorectal cancer (EOCRC), health history, and risk factors for colorectal cancer (CRC); Table S3: Characteristics of colorectal cancer (CRC) diagnosis among respondents; Table S4: Treatment experience of early age onset colorectal cancer (EOCRC) patients; Table S5: Mental health experience of early age onset colorectal cancer (EOCRC) patients; Table S6: Mental health experience of caregivers; Table S7: Financial health among patients and caregivers; Table S8: Clinical trial experience of early age onset colorectal cancer (EOCRC) patients.
