# Supplementary material for: A Survey Detailing Early Onset Colorectal Cancer Patient and Caregiver Experiences in Canada
Source: Curr Oncol. 2024 May 31;31(6):3149–60. doi: 10.3390/curroncol31060238 (PMC11203371; doi:10.3390/curroncol31060238)
Supplement: Supplementary file 1 [file curroncol-31-00238-s001.zip › curroncol-2975865-supplementary.pdf]

Supplementary Materials

Supplementary Material 1: Survey Form

Never Too Young - Patient & Caregiver Experiences with Early Onset Colorectal Cancer in Canada Survey 2022

PATIENT INFORMATION SHEET

Patient & Caregiver Survey

(Patient & Caregiver Experiences with Early Onset Colorectal Cancer in Canada)

Survey Sponsor: Colorectal Cancer Canada

Principal Investigator: Dr. Rebecca Auer

Please read this Patient Information Sheet carefully before you decide if you would like to participate in this survey.

Participation is voluntary and completion of the survey implies your consent to participate in this survey.

Why am I being given this form?
You are being asked to participate in this survey because you are a patient with early age onset of colorectal cancer (EOA –CRC), diagnosed with CRC between the ages of 18-50, or you are a caregiver of an EOA-CRC patient.

Why is this survey being done?
Colorectal Cancer Canada's Never Too Young (N2Y) program was established in 2018 and aims to increase public awareness around early-age onset of the disease in Canada, to support patients throughout their care pathway, and to sensitize the healthcare community about the unique challenges that these patients face.

The goal of our first Never Too Young, Early-Age Onset Experiences Survey completed in 2020 was to gain additional insights into the experiences of early-age onset patients in Canada, from symptoms to diagnosis, to outcomes and quality of life. The results of that survey enabled Colorectal Cancer Canada to build an early age onset toolkit for the benefit of Canadian patients. This tool kit is available at (https://www.colorectalcancercanada.com/app/uploads/2021/12/N2Y-Patient-and-Caregiver-Toolkit.pdf).

This second survey further explores the perceptions of EOA-CRC patients as well as their caregivers regarding their experiences and needs related to a colorectal cancer diagnosis. The findings of this survey will be published in a survey report, with the aim of providing Canadian patients, caregivers, practitioners, and researchers with additional data and insight on the experiences of EOA-CRC patients in Canada, with the goal of improving patient outcomes and their quality of life.

What is expected of me?
This is a voluntary process where you will be completing the survey through an electronic link. You may answer as many questions as you like, however by completing the entire survey, we will be better informed to help patients.

How long will I be involved in the survey?
This Survey is expected to take 25-30 minutes to complete. We anticipate that it will take approximately 6 months to collect completed surveys from 500 patients/ caregivers across Canada.

What are the potential risks of participation?
There are no risks to participating in this survey. You can choose not to respond to any of the questions in the survey.

How can I expect to benefit from participating in this survey?

This study is not likely to benefit you directly. However, the results from this survey will enable us to better understand patient experiences in terms of symptoms, diagnosis, and the impact of treatment on their quality of life.

The interpretation and review of the results of this survey (without any patient identification) will be submitted to a scientific journal for publication with the goal of prioritizing research of EOA-CRC in Canada. The information we gather from the survey will be available to help develop further clinical trials for this patient population.

Do I have to participate? What alternatives do I have? If I agree now, can I change my mind and withdraw later?

Your participation in this survey is voluntary, and you may choose to decline participation. Your willingness to be involved with this survey will not affect your care in any way. The survey is anonymous, therefore once submitted, we cannot withdraw your responses from the database.

Will there be any additional costs to me or compensation for my participation?

There are no costs associated with your participation in this survey. You will not be financially compensated for your participation however, to show our appreciation for the time spent completing the survey, 5 of the total respondents will randomly be selected to receive a $50 Lululemon E-Gift Card. How is

my personal information being protected?

• All information collected from this survey will be anonymous. We will not collect any personal identifiers such as name, address, date of birth, hospital ID number, etc. However, we will be collecting email addresses on an optional basis if you are interested in the random selection for the gift card and/or receiving any additional information from Colorectal Cancer Canada.

• For audit purposes only, the anonymous, completed surveys may be reviewed under the supervision of Dr. Auer's research staff by representatives from:

o The Ontario Cancer Research Ethics Board (OCREB),

o The Ottawa Hospital Research Institute (OHRI)

o Colorectal Cancer Canada (CCC)

• Survey records will be kept for 10 years, after this time they will be destroyed.

Whom do I contact if I have any further questions?

If you have any questions regarding the survey or Colorectal Cancer Canada's early age onset program called the Never Too Young (N2Y) Program, please contact the Never Too Young Program Manager, Uditi Ratti, at uditir@colorectalcancercanada.com

If you have any questions about your rights as a participant in this survey, you may contact the Office of the Chair of the Ontario Cancer Research Ethics Board at 416-673-6648 or Toll Free: 1-866-678- 6427 ext. 6648

Never Too Young - Patient & Caregiver Experiences with Early Onset Colorectal Cancer in Canada Survey 2022

Thank you for participating in our survey. Your feedback is important.

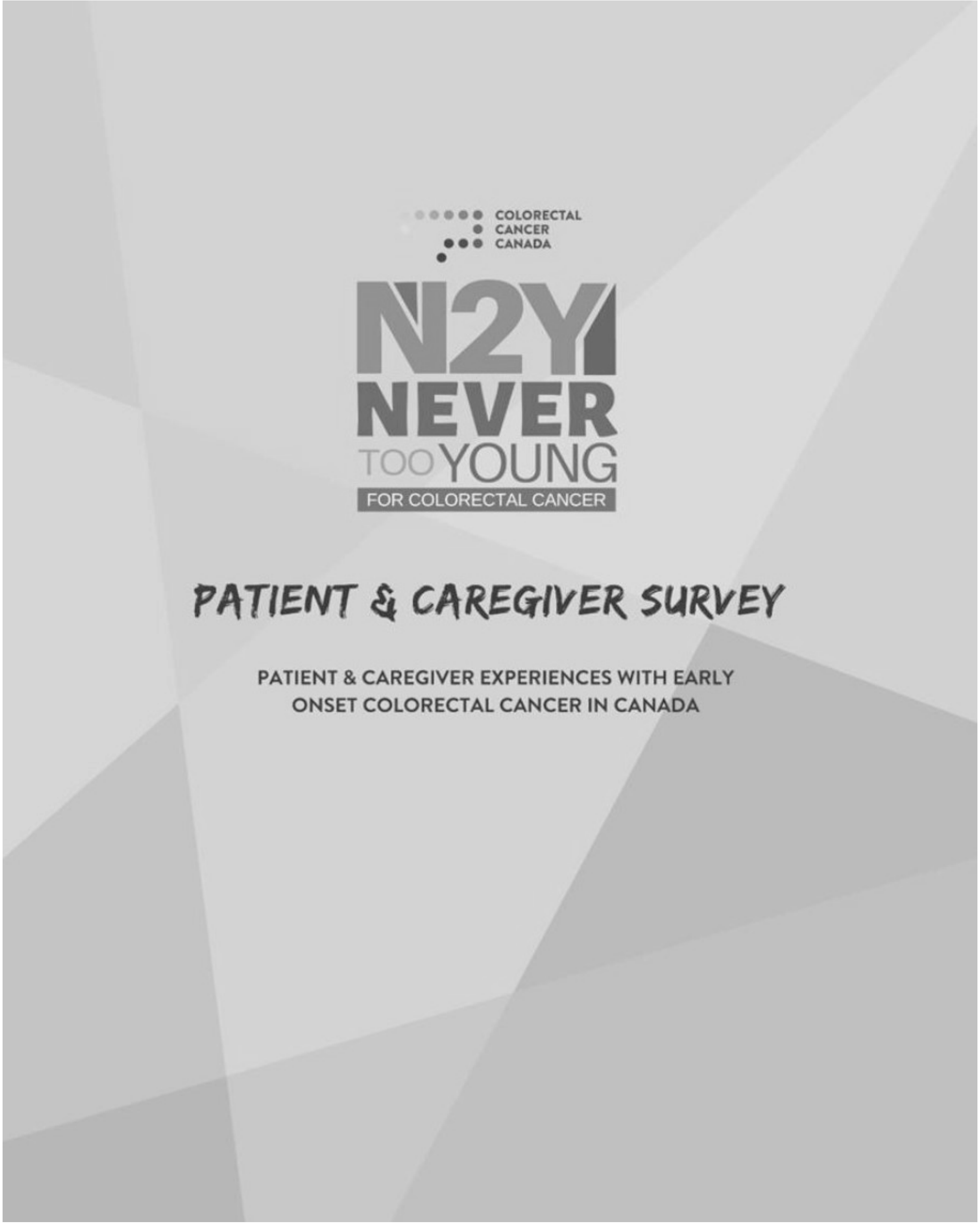

Never Too Young - Patient & Caregiver Experiences with Early Onset Colorectal Cancer in Canada Survey 2022

Patient & Caregiver Experiences with Early Onset Colorectal Cancer in Canada

Purpose

Colorectal Cancer Canada's Never Too Young program was established in 2018 and aims to increase awareness around early-age onset colorectal cancer (EAO CRC) in Canada, to support patients throughout their journey, and to sensitize the healthcare community about the unique challenges that patients face. The goal of the Never Too Young: Early-Age Onset Experiences survey was to gain additional insight into the experiences of early-age onset patients in Canada, from symptoms to diagnosis to outcomes and quality of life. This is our second survey to explore perceptions of early age onset colorectal cancer patients fifty (50) years of age and younger as well as their caregivers regarding their experiences and needs related to a cancer diagnosis. The results of our first survey enabled us to build an early age onset toolkit for the benefit of Canadian patients. The findings of this survey will be published in a survey report, with the aim of providing patients, caregivers, practitioners, and researchers with additional data and insight on the experiences of early-age onset colorectal cancer patients in Canada, which will hopefully aid in improving outcomes and the quality of life of future colorectal cancer patients in Canada.

https://www.colorectalcancercanada.com/app/uploads/2021/12/N2Y-Patient-and-Caregiver-Toolkit.pdf

Instructions

Participation in this study is entirely voluntary. You can choose not to participate or discontinue participation at any time.

- **The survey includes three main sections: Demographics (e.g., age, gender, race) and lifestyle.**
- **Your experience with symptoms and diagnosis; and**
- **Your treatment and post-treatment experience.**

If you are a caregiver, you will be automatically forwarded to the specific caregiver-based sections.

This survey should take approximately 25-30 minutes to complete. Once you complete the questions and submit the survey, your response will be sent directly to the Colorectal Cancer Canada Never Too Young Program Manager.

All your responses to this survey will remain anonymous and cannot be linked to you in any way. Once you submit your completed survey, there will be no way to withdraw your responses from the study because there is no mechanism to identify you.

Study data are returned to the Program Manager in a digital format that does not

identify individual responses. The digital, non-identifiable, data will be kept by the Program Manager on a password-protected computer. Combined or analyzed, non-identifiable data will be shared with the medical community and the public to increase awareness about this important public health issue.

There are no risks associated with this study. While you will not experience any direct benefits from participation, information collected in this study may benefit others in the future by contributing to our knowledge and understanding of early onset colorectal cancer patient and caregiver experiences in Canada.

If you have any questions regarding the survey or Colorectal Cancer Canada's early age onset program called the Never Too Young (N2Y) Program, please contact the Never Too Young Program Manager, Uditi Ratti, at uditir@colorectalcancercanada.com

To show our appreciation for the time spent completing the survey, 10 of the total respondents will randomly be selected to receive a $50 Lululemon E-Gift Card. To opt in, please leave your mailing address in the appropriate field.

If you agree to participate, you are asked to fill out the survey provided below. By completing and submitting this survey, you are indicating your consent to participate in this study and to the eventual publication of the analysis of the results.

Never Too Young - Patient & Caregiver Experiences with Early Onset Colorectal Cancer in Canada Survey 2022

1. What is your connection to colorectal cancer?

◯ Patient undergoing treatment

◯ Patient not undergoing treatment ◯

No Evidence of Disease (NED)

◯ Caregiver (If you click Caregiver, click on next, this shall take you to the Caregiver section)

Patient: Demographics

2. How old are you now?

◯ Under 20

◯ 20-29

◯ 30-39

◯ 40-49

◯ 50-59

◯ 60+

3. What is your race / ethnicity?

[▼]

[ ]

4. What is your gender?

◯ Female

◯ Male

◯ Transgender ◯

Intersex

◯ Non-Binary

◯ Prefer not to say Prefer

◯ to self describe

[ ]

5. What is your marital status?

- Single
- Married / Common Law
- Divorced
- Other (please specify)

6. Do you have children?

- Yes
- No

7. What is the highest degree or level of education you have completed?

- Less than high school diploma
- High school degree or equivalent
- Cégep / college diploma/trade college
- Bachelor's degree
- Master's degree
- Doctorate
- Other (please specify)

8. Select your province of residence.

- Alberta
- British Columbia
- Manitoba
- New Brunswick
- Newfoundland & Labrador
- Northwest Territories
- Nova Scotia
- Nunavut
- Ontario
- Prince Edward Island
- Quebec
- Saskatchewan
- Yukon

9. What best describes where you live?

- Urban
- Suburban
- Rural

10. Are you currently working?

- Yes
- No
- Prefer not to answer

Never Too Young - Patient & Caregiver Experiences with Early Onset Colorectal Cancer in Canada Survey 2022

Patient: CRC Awareness Prior to Diagnosis

11. Prior to diagnosis, were you aware colorectal cancer can happen in people younger than 50?

- Yes
- No
- Vaguely

12. Did you know the signs or symptoms of colorectal cancer BEFORE being diagnosed?

- Yes
- No
- Vaguely

13. Did you discuss your family health history, including colorectal cancer risk factors, with your family/friends or doctor BEFORE your diagnosis?

- Yes, with my family and/or friends
- Yes, with my doctor
- Yes, with my family and/or friends AND my doctor
- I did not discuss colorectal cancer risk factors with my family, friends or my doctor

Never Too Young - Patient & Caregiver Experiences with Early Onset Colorectal Cancer in Canada Survey 2022

Patient: Details of your Colorectal Cancer

14. What year were you diagnosed with colorectal cancer?

15. How old were you when you were diagnosed with colorectal cancer?

- 45-50
- 40-44
- 35-39
- 30-34
- 25-29
- 20-24
- Younger than 20

16. What type of cancer were you diagnosed with (primary tumor)?

- Colon
- Rectal
- I don't know

17. What side was your primary tumor located on?

Right sided is defined as cancer of the cecum and the ascending colon up to the hepatic flexure. Left-sided colon cancer is defined as cancer of the splenic flexure and cancer in regions distal to the splenic flexure, including the rectum.
(Source: Right-Sided vs Left-Sided Colorectal Cancer – Hematology & Oncology (hematologyandoncology.net)

- Left
- Right
- I don't know

18. What stage colorectal cancer were you diagnosed with?

Stage 0 - the tumour is usually small and has not spread outside the inner lining,

Stage l – the tumour has invaded the layers of the colon or rectum but has not grown outside the walls of the colon or into the surrounding tissue.

Stages II – the tumour has grown through the walls of the colon or rectum into nearby tissue but has not affected lymph nodes.

Stage III - the tumor is larger and has spread outside the colon or rectum to nearby tissues but not to distant organs,

Stage IV (metastatic) - the cancer has spread through blood or lymphatic system to a distant site in the body most commonly the liver, lungs, or peritoneum

- ◯ 0
- ◯ I
- ◯ II
- ◯ III
- ◯ IV (metastatic) I
- ◯ don't know

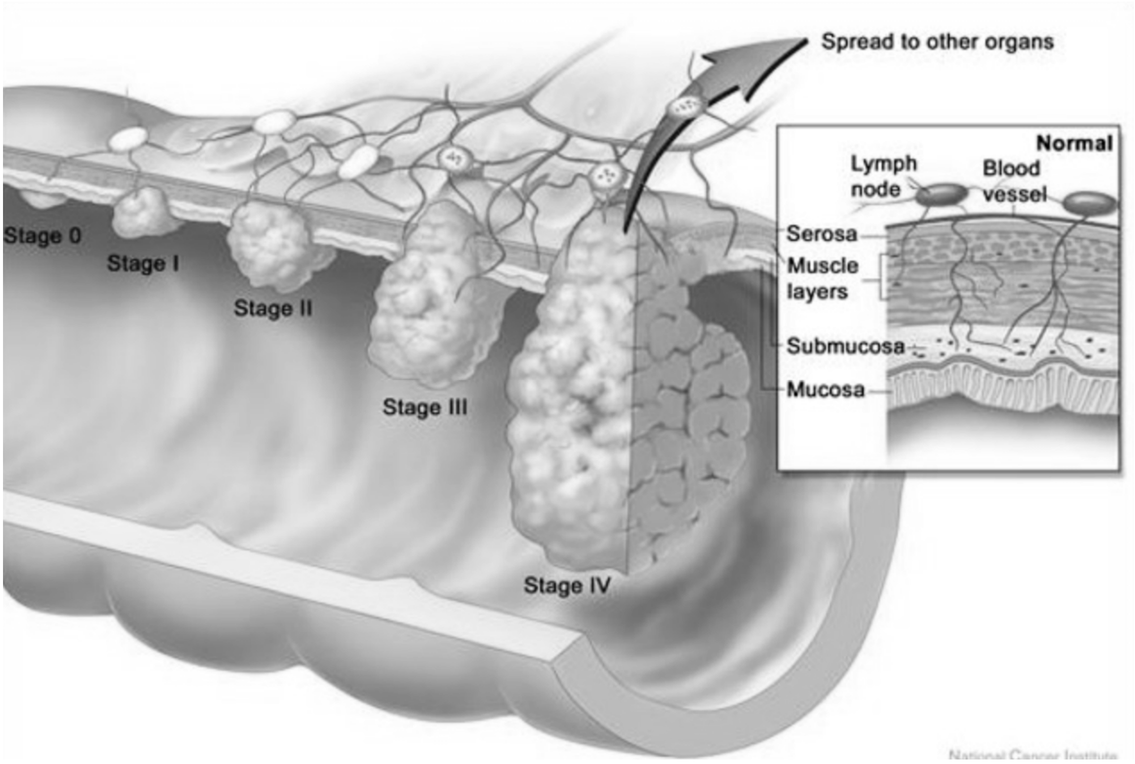

19. If applicable, please select locations of your metastases:

Check all that apply

- [ ] Lung
- [ ] Liver
- [ ] Peritoneum
- [ ] Lymph nodes
- [ ] Bone
- [ ] Spine
- [ ] Brain
- [ ] Prefer not to answer
- [ ] N/A
- [ ] Other (please specify)

20. If applicable, how long have you been NED (No Evidence of Disease)?

- ( ) Less than 1 year
- ( ) 1-2 years
- ( ) 2-3 years
- ( ) 3-4 years
- ( ) 4-5 years
- ( ) 5-6 years
- ( ) 6-7 years
- ( ) 7-8 years
- ( ) 8-9 years
- ( ) 9-10 years
- ( ) Longer than 10 years
- ( ) N/A

21. If you have had a recurrence, how many recurrences have you had?

- ( ) 1
- ( ) 2
- ( ) 3
- ( ) 4
- ( ) 5
- ( ) More than 5
- ( ) N/A

Patient: Risk Factors

22. Do you have a family history of colorectal cancer in first or second-degree relatives?

A first-degree relative is a close blood relative (full siblings, parents and children).

A second-degree relative is a more distant blood relative (grandparents, nieces, nephews, aunts, uncles, half-siblings).

- ◯ Yes
- ◯ No
- ◯ I don't know

23. Prior to diagnosis, did you have a personal history of the following?

Check all that apply

- ☐ Colitis
- ☐ Crohn's
- ☐ IBD/IBS
- ☐ Adenomatous polyps
- ☐ GI cancers
- ☐ Endometrial cancer
- ☐ Ovarian cancer
- ☐ Breast cancer
- ☐ Prostate cancer
- ☐ No history
- ☐ I don't know

24. Do you have a family history of the following?

Check all that apply

- ☐ Colitis
- ☐ Crohn's
- ☐ IBD/IBS
- ☐ Adenomatous polyps
- ☐ GI cancers
- ☐ Endometrial cancer
- ☐ Ovarian cancer
- ☐ Breast cancer
- ☐ Prostate cancer
- ☐ No history
- ☐ I don't know

25. What was your approximate weight (pounds) at diagnosis?

[ ]

26. What is your height (feet and inches)?

[ ]

27. Were you a smoker at the time of your diagnosis, if yes, about how many packs of cigarettes would your smoke per week?

Mark only one circle

- ○ No
- ○ Yes
- ○ If yes, then how many packs per week?

[ ]

28. Were you ever a smoker in the past? If yes, about how many years had you been a regular smoker?

Mark only one circle.

- ○ No
- ○ Yes
- ○ If yes, then how many years of smoking?

[ ]

29. Did you regularly consume alcohol prior to your diagnosis? If yes, how many drinks per week did you consume on average?

Mark only one circle.

- ◯ No
- ◯ Yes
- ◯ If Yes (how many drinks per week)

30. Did you regularly consume sugar-sweetened beverages prior to your diagnosis (e.g., soda).
Mark only one circle.

- ◯ Frequently
- ◯ Occasionally
- ◯ Rare to never

31. How would you rate your level of physical activity prior to diagnosis?

Sedentary = less than 30 minutes of moderate/vigorous activity per week,

Lightly active = 30 - 60 minutes of moderate/vigorous activity per week,

Moderately active = 60 - 150 minutes of moderate/vigorous activity per week,

Highly active = 150 + minutes of moderate/vigorous activity per week.

Moderate-intensity aerobic activity makes you breathe harder and your heartbeat faster. You should be able to talk, but not sing. Vigorous-intensity aerobic activity makes your heart rate increase quite a bit and you won't be able to say more than a few words without needing to catch your breath. (Source: Physical Activity Tips for Adults (18-64 years) - Canada.ca)

- ◯ Sedentary
- ◯ Lightly active
- ◯ Moderately active
- ◯ Highly active

32. Did you follow any of the following diets prior to your diagnosis (choose all that apply)?

Check all that apply

- [ ] No specific diet
- [ ] Dairy-free
- [ ] Gluten-free
- [ ] Vegetarian
- [ ] Vegan
- [ ] Mediterranean diet
- [ ] Keto Pescatarian Prefer
- [ ] not to answer Other
- [ ] (please specify)

33. On average, how many servings of Vegetables (including fresh and frozen) did you eat per day prior to your diagnosis?

One serving = 125 mL (½ cup) fresh, frozen or canned vegetables, 250 mL (1 cup) raw leafy veggies or salad (Source: Canada's Food Guide).

Mark only one circle

- ( ) 0 Servings
- ( ) 1 Serving
- ( ) 2 Servings
- ( ) 3 Servings
- ( ) 4 Servings
- ( ) 5 Servings
- ( ) 6 Servings
- ( ) 7 Servings
- ( ) 8 Servings
- ( ) More than 8 Servings

34. On average, how many servings of Fruits (including fresh and frozen) did you eat per day prior to your diagnosis?

One serving = 125 mL (½ cup) fresh, frozen or canned vegetables, 250 mL (1 cup) raw leafy veggies or salad (Source: Canada’s Food Guide).

Mark only one circle

- ◯ 0 Servings
- ◯ 1 Serving
- ◯ 2 Servings
- ◯ 3 Servings
- ◯ 4 Servings
- ◯ 5 Servings
- ◯ 6 Servings
- ◯ 7 Servings
- ◯ 8 Servings
- ◯ More than 8 Servings

Patient: Symptoms

35. Which of the following symptom(s) appeared first?
Check all that apply.

- [ ] No symptoms
- [ ] Diarrhea
- [ ] Constipation
- [ ] Weight loss
- [ ] Blood in stool
- [ ] Rectal bleeding
- [ ] Weakness and/or fatigue
- [ ] Persistent cramps
- [ ] Bowel never feels empty
- [ ] Bloating and/or gas
- [ ] Nausea and/or vomiting Pain Other
- [ ] (please specify)

36. Which of the following symptom(s) appeared subsequently?
Check all that apply.

- [ ] No symptoms
- [ ] Diarrhea
- [ ] Constipation
- [ ] Weight loss
- [ ] Blood in stool
- [ ] Rectal bleeding
- [ ] Weakness and/or fatigue
- [ ] Persistent cramps
- [ ] Bowel never feels empty
- [ ] Bloating and/or gas
- [ ] Nausea and/or vomiting Pain Other
- [ ] (please specify)

37. At what age (years) did you first notice symptoms?

38. Which doctors did you see once you noticed your symptoms?
Check all that apply.

- [ ] Primary care provider
- [ ] Pediatrician
- [ ] Gastroenterologist
- [ ] Emergency Room Doctor
- [ ] OBGYN
- [ ] Urologist
- [ ] Urgent care
- [ ] Other (please specify)

39. How long did you experience symptoms before going to the doctor?
Mark only one circle

- ◯ Less than 1 month ◯
- 1-3 months
- ◯ 3-6 months
- ◯ >6 months

40. How many different doctors did you see before you were diagnosed with colorectal cancer?
Mark only one circle

- ◯ 1
- ◯ 2
- ◯ 3
- ◯ 4
- ◯ 5
- ◯ More than 5 doctors

41. In total, about how many appointments did you have until one of your doctors suspected colorectal cancer?
Mark only one circle

- ◯ 1
- ◯ 2
- ◯ 3
- ◯ 4
- ◯ 5
- ◯ 6
- ◯ 7
- ◯ 8
- ◯ 9
- ◯ 10
- ◯ More than 10 appointments

42. At any point before diagnosis did a doctor perform a rectal exam?
Mark only one circle

- ◯ Yes
- ◯ No
- ◯ N/A

43. Which doctor did you see that led to your colonoscopy?
Check all that apply.

- ☐ Family/primary care provider
- ☐ Gastroenterologist Emergency
- ☐ Room Doctor OBGYN
- ☐ Urgent care
- ☐ Urologist
- ☐ Pediatrician
- ☐ Surgeon
- ☐ Other (please specify)

[ ]

44. How long did it take to be diagnosed with colorectal cancer after you first sought medical attention for your symptoms?
Mark only one circle .

- ◯ Less than 1 month
- ◯ 1-3 months
- ◯ 3-6 months
- ◯ Longer than 6 months

45. How concerned were the doctors when you described your signs and symptoms?
Mark only one circle.

◯ They did not seem concerned ◯

They seemed slightly concerned

- ◯ They seemed moderately concerned
- ◯ They seemed very concerned

46. Do you feel your doctors dismissed the signs and symptoms of colorectal cancer due to your age?
Mark only one circle.

- ◯ Yes
- ◯ No
- ◯ I don't know
- ◯ Yes, but for a reason other than age.

47. Do you believe you were MISTAKENLY diagnosed with any of these conditions prior to your diagnosis of colorectal cancer?
Check all that apply.

- ☐ Hemorrhoids Symptoms
- ☐ ofchildbirth
- ☐ Appendicitis
- ☐ Gynecological issues IBS
- ☐ IBD
- ☐ Mental health issues
- ☐ Crohn's/Colitis
- ☐ Other (please specify)

____

- ☐ None of the above

Patient: Diagnosis

48. Which doctor did you see after diagnosis?
Check all that apply.

- [ ] Family/primary care provider
- [ ] Gastroenterologist Oncologist
- [ ] Pediatrician
- [ ] Surgeon
- [ ] Other (please specify)

49. Did you have genetic testing before or after being diagnosed with colorectal cancer?

- ◯ Yes, before diagnosis
- ◯ Yes, after diagnosis
- ◯ No
- ◯ I don't know

50. If yes, did you test positive for genetic variant, or syndrome such as Lynch Syndrome?

- ◯ No
- ◯ Yes
- ◯ If yes, (Signify variant /syndrome if known)

51. Will you have your family tested for genetic variants related to colorectal cancer?

- ◯ Yes
- ◯ No
- ◯ I don't know

52. Were you informed by your medical team that your children and first-degree relatives should be screened 10 years younger than the age at which you were diagnosed?

- ◯ Yes
- ◯ No

53. Did you have surgery before consulting with an oncologist?

- Yes
- No
- I don't know

54. Did you seek a second opinion with a doctor about treatment options?

- Yes
- No

55. Did you understand and feel fully informed about your treatment plan and side effects when it was explained to you by your doctor?

- Yes
- No
- I don't know

Patient: Biomarkers & Treatment

Biomarkers, short for biological markers, are biological molecules found in blood, other body fluids or tissues that may provide information about a tumour. Results of a biomarker test can help create a more personalized approach to cancer care that will allow you to make more informed decisions.

56. Was biomarker testing a part of your early age onset colorectal cancer treatment care path?

Select "yes" if biomarker testing was introduced to you, even if you did not go ahead with testing. If biomarker testing was not a part of your treatment care path, select "no" and you will be redirected to the next section of the survey

- ◯ Yes
- ◯ No (Click Next and Skip to question 63)
- ◯ I don’t know (Click Next and Skip to question 63)

57. Did your oncologist or any other member of your medical team explain biomarker testing (or tumour profiling) before treatment started?

- ◯ Yes
- ◯ No
- ◯ I don't know

58. Have you heard of biomarkers from another source?

- ◯ Yes
- ◯ No

59. If yes, how did you learn about biomarkers?

[ ]

60. Have you had your tumour(s) tested for biomarkers?

- ◯ Yes
- ◯ No
- ◯ I don't know

61. If yes, do you know the results of your biomarker testing?
Check all that apply.

- ☐ MSI-H or deficient MMR
- ☐ MSS or proficient MMR
- ☐ K-RAS mutant
- ☐ K-RAS wild type
- ☐ N-RAS mutant N-
- ☐ RAS wild type
- ☐ BRAF mutant
- ☐ HER2
- ☐ NTRK
- ☐ PIK3CA
- ☐ BRCA1
- ☐ N/A
- ☐ I don't know
- ☐ Other (please specify)

62. If you had your biomarkers tested, did your doctor use the results to help select a treatment that is best for you?

- ◯ Yes
- ◯ No
- ◯ I don't know
- ◯ N/A

Patient: Clinical Trials

63. Were clinical trials a part of your early age onset colorectal cancer care path?

Select "yes" if clinical trials were introduced as an option, even if you did not participate in a clinical trial. If clinical trials were not a part of your care path, select "no" and you will be redirected to the next section of the survey

- Yes
- No (Click Next and Skip to Question 76)
- I don't know (Click Next and Skip to Question 76)

64. Did your doctor mention clinical trials at diagnosis?

- Yes
- No
- I don't remember

65. Did you hear about clinical trials from another source?

- Yes
- No

66. If yes, how did you hear about clinical trials?

67. Did you feel sufficiently informed about clinical trials?

- Yes
- No

68. Would you like to learn more about clinical trials?

- Yes
- No

69. How important do you believe clinical trials are as treatment options for young-onset colorectal cancer patients?

- Very important
- Somewhat important
- Not important

70. Would you consider participating in a clinical trial?

- Yes
- No

71. If not, please specify why not:

72. If yes, please indicate how strongly you agree or disagree with the following:

	Strongly Disagree	Disagree	Neutral	Agree	Strongly Agree
Improve my own condition					
Contribute to advances in knowledge and treatment					
Access to treatments that are not widely available					
Receive free medical care					
Reduce wait times for seeing specialists					

73. Please indicate how strongly you agree or disagree with the following:
Mark only one circle per row:

	N/A	Strongly Disagree	Disagree	Neutral	Agree	Strongly Agree
Searching for a clinical trial was easy	○	○	○	○	○	○
Searching for a clinical trial was overwhelming	○	○	○	○	○	○
It was easy to understand the patient information of the clinical trial	○	○	○	○	○	○
It was difficult to understand the patient information of the clinical trial	○	○	○	○	○	○
My medical team strongly encouraged me to participate in a clinical trial	○	○	○	○	○	○
My medical team was not interested in clinical trials	○	○	○	○	○	○

74. Did you participate in a clinical trial?

- ○ Yes
- ○ No
- ○ Prefer not to answer

75. If you would like, please share your experience with clinical trials.

[ ]

Patient: Treatment

76. Were you a candidate for surgery at initial diagnosis?

- Yes
- No
- Prefer not to answer

77. If yes, what type of surgery?

- Right hemicolectomy
- Sigmoid colectomy
- Left hemicolectomy
- Lower anterior resection
- Other (please specify)

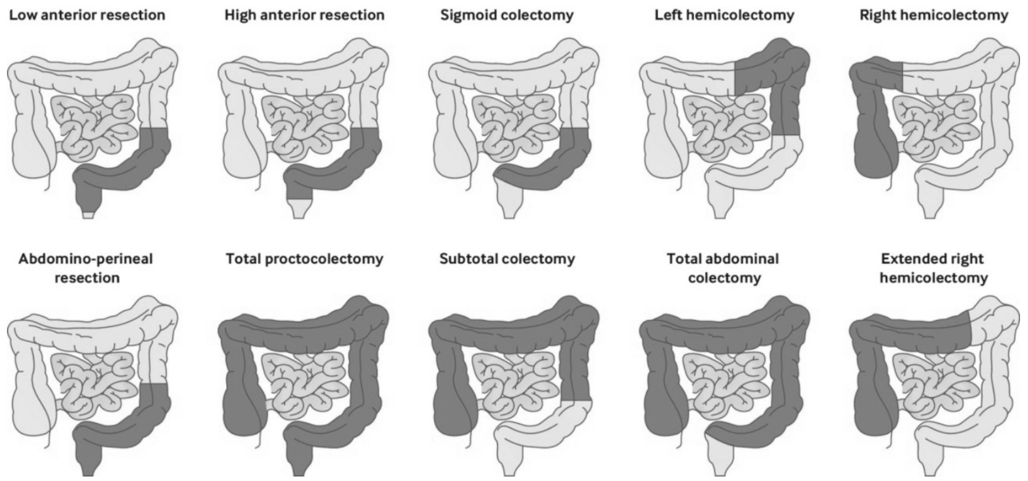

78. Did you have radiation?

- Yes
- No
- Prefer not to answer

79. Following your surgery, did you have a colostomy or ileostomy?

- ◯ Yes
- ◯ No
- ◯ Prefer not to answer
- ◯ N/A

80. If yes, please rate your experiences below.
Please mark only one circle per row -

	Never	Sometimes	Often	Always	Prefer not to answer
Have you had a leakage of stools from your back passage?	◯	◯	◯	◯	◯
Have you had sore skin around your anal area?	◯	◯	◯	◯	◯
Did frequent bowel movements occur during the night?	◯	◯	◯	◯	◯
Did you feel embarrassed because of your bowel movement?	◯	◯	◯	◯	◯
Have you had leakage of stools from your stoma bag?	◯	◯	◯	◯	◯
Have you had sore skin around your stoma?	◯	◯	◯	◯	◯
Did you have problems caring for your stoma?	◯	◯	◯	◯	◯
Has your ostomy interfered with your social activities?	◯	◯	◯	◯	◯
Has your ostomy interfered with your recreational / sports activities?	◯	◯	◯	◯	◯
Has your ostomy interfered with your personal relationships?	◯	◯	◯	◯	◯
Has your ostomy interfered with your ability to be intimate?	◯	◯	◯	◯	◯

Patient: Challenges

81. Did any of your providers discuss sexual side effects due to radiation and surgery before treatment began?

- [ ] Painful sex
- [ ] Decrease in sex drive
- [ ] Loss of sexual function
- [ ] Infertility
- [ ] I don't remember
- [ ] Providers did not discuss any of the above

82. Did you experience any of the following after your treatment?

- [ ] Painful sex
- [ ] Decrease in sex drive
- [ ] Loss of sexual function
- [ ] Infertility
- [ ] I don't remember
- [ ] Prefer not to answer

83. Please rate your level of sexual function issues after treatment and surgery.

- ( ) No loss of sexual function
- ( ) Partial loss of sexual function
- ( ) Total loss of sexual function ( )

Prefer not to answer

84. Have you received any guidance from medical staff regarding sexual health?
Check all that apply.

- [ ] No Guidance Doctors
- [ ] mentionedit
- [ ] Specialist referral
- [ ] Materials Provided

85. Please indicate the impact of the following issues:

Please mark only one circle per row -

	Always	Often	Sometimes	Never	Prefer not to Answer
Even with guidance from medical staff, I am still afraid to have sex	○	○	○	○	○
My sexual dysfunction puts strain on my relationships	○	○	○	○	○
My sexual dysfunction affects my ability to become intimate with others	○	○	○	○	○
I feel awkward to receive guidance for ○ sexual life	○	○	○	○	
I believe I am not a complete person due to sexual dysfunction	○	○	○	○	○
I worry I am not 'enough' for a significant other due to sexual dysfunction	○	○	○	○	○

86. Did a medical professional talk to you about fertility preservation at any point during diagnosis or treatment?

Mark only one circle

- ○ Yes
- ○ No
- ○ Prefer not to answer
- ○ N/A

87. Did you have reproductive health issues (fertility) before treatment?

Mark only one circle

- ○ Yes
- ○ No
- ○ Prefer not to answer
- ○ N/A

88. Has treatment left you infertile or sterile?
Mark only one circle

- ◯ Yes
- ◯ No
- ◯ Prefer not to answer
- ◯ N/A

89. If you like, please share your experience with changes in sexual or reproductive health due to treatment.

Patient: Mental Health

90. Please rate your experiences while undergoing treatment below.

Please mark only one circle per row -

	Never	Sometimes	Often	Always	Prefer not to answer
I feel / felt emotionally exhausted					
I have withdrawn / withdrew from other people					
I feel / felt like I need / needed help for my depression					
I feel satisfied with how I am coping with my illness					
I am able to enjoy life					
I am afraid that my cancer may recur					
Thoughts of recurrence interfere with my daily activities					
I am / was concerned about my mental health					
I feel / felt sudden feelings of panic and anxiety					
How often did you have to limit social activities due to fatigue?					
How often did your fatigue make it difficult to make decisions?					
How often were you too tired to do everyday activities?					
How much do / did you worry about pain?					
How much does / did pain interfere with your family and day - to - day life?					

Never Too Young - Patient & Caregiver Experiences with Early Onset Colorectal Cancer in Canada Survey 2022

Patient: Financial Experience

91. What was your employment status at the time of diagnosis?

Mark only one circle

- Employed
- Unemployed
- Retired
- Prefer not to answer
- Other (please specify)

92. Did you take a leave of absence, quit a job or leave school because of diagnosis?

- Yes
- No

If you left your job or took a leave did you have private employment insurance coverage.

93. Was the Canada Employment Sickness Benefit sufficient?

- Yes
- No
- Prefer not to answer
- N/A

94. Did you have private employment insurance coverage?

- Yes
- No
- Prefer not to answer
- N/A

95. Did your employer/school help accommodate your treatment schedule?

- Yes
- No
- Prefer not to answer
- N/A

96. Please rate your experiences below

Mark only one circle per row -

	Strongly Disagree	Disagree	Neutral	Agree	Strongly Agree	Prefer not to answer
I feel financially stressed	◯	◯	◯	◯	◯	◯
I worry about the loss of my family's financial stability because of the cost of cancer care	◯	◯	◯	◯	◯	◯
I worry about the financial problems I will have in the future due to my illness / treatment	◯	◯	◯	◯	◯	◯
I worry that my public insurance is not adequate enough to cover my cost of cancer care and treatment	◯	◯	◯	◯	◯	◯
I feel in control of my financial situation	◯	◯	◯	◯	◯	◯
I am optimistic about my financial future	◯	◯	◯	◯	◯	◯

Patient: Impact of Covid - 19

97. Were you diagnosed/did you receive treatment after the initial outbreak of the Covid-19 pandemic?

- ◯ Yes
- ◯ No (Click next and Skip to Question 109)

98. What precautions did you take to reduce your risk when attending medical appointments?

Check all that apply.

- ☐ Surgical Gown
- ☐ Face Mask
- ☐ Gloves
- ☐ Glasses/ Googles
- ☐ Hand Washing
- ☐ Other (please specify)

99. Due to Covid-19, was there a delay in your screening for colorectal cancer?

- ◯ No
- ◯ I don't know
- ◯ N/A
- ◯ Yes
- ◯ If yes for how long?

100. Due to Covid-19, was there a delay in your cancer imaging?

- ◯ No
- ◯ I don't know
- ◯ N/A
- ◯ Yes
- ◯ If yes for how long?

101. Due to Covid-19, was there a delay with your surgery?

- Yes
- No
- I don't know
- N/A

102. Due to Covid-19, was there a delay with your radiation therapy?

- Yes
- No
- I don't know
- N/A

103. Due to Covid-19, was there a delay with your cancer treatment (chemotherapy, immunotherapy)?

- No
- I don't know
- N/A
- Yes
- If yes, for how long?

104. Due to Covid-19, did you have appointments done virtually?

- No
- I don't know
- N/A
- Yes
- If yes, how much would you rate your experience of virtual appointment out of 10?

105. If yes, would you have preferred to have had your appointment(s) in person?

- Yes
- No
- I don't know
- N/A

106. Due to Covid - 19, which of these factors has made an impact on your access to cancer care? Check all that apply.

- [ ] No changes in care
- [ ] Care Delayed
- [ ] Location of care switched
- [ ] Treatment switched Other
- [ ] (please specify)

107. Due to Covid-19, was there a delay in your clinical trial?

- ( ) Yes
- ( ) No
- ( ) I don't know
- ( ) N/A
- ( ) Yes (if so for how long?)

108. Has Covid - 19 affected your social determinants of health?
Check all that apply.

- [ ] Access to transportation
- [ ] Access to healthcare clinics / hospitals
- [ ] Difficulties getting childcare
- [ ] Job Loss
- [ ] Access to adequate food
- [ ] Access to pharmacies / acquiring medications
- [ ] Other (please specify)

Patient: Summary

109. What is the biggest challenge you've faced as someone diagnosed under 50?

110. The patient survey questions have now ended. Please select "finished" to proceed to the conclusion section.

Finished (skip to question 200).

Caregiver: Demographics

111. What is your race / ethnicity is / was your loved one ?

112. What gender does / did your loved one most identify with?

- Female
- Male
- Transgender
- Intersex
- Non-Binary
- Prefer not to say
- Prefer to self describe

113. What was your loved one's marital status at the time of the diagnosis?

- Single
- Married / Common Law
- Divorced
- Other (please specify)

114. Did your loved one have children at the time of diagnosis?

- Yes
- No

115. What is the highest degree or level of education your loved one has completed?

- Less than high school diploma
- High school degree or equivalent
- Cégep / college diploma/trade college
- Bachelor's degree
- Master's degree
- Doctorate
- Other (please specify)

116. Select your loved one's province of residence.

- Alberta
- British Columbia
- Manitoba
- New Brunswick
- Newfoundland & Labrador
- Northwest Territories
- Nova Scotia
- Nunavut
- Ontario
- Prince Edward Island
- Quebec
- Saskatchewan
- Yukon

117. What best describes where your loved one lived at the time of the diagnosis?

- Urban
- Suburban
- Rural

118. If applicable, is your loved one currently working?

- Yes
- No
- Prefer not to answer

Caregiver: CRC Awareness Prior to Diagnosis

119. Prior to their diagnosis, was your loved one aware that colorectal cancer can happen in people younger than 50?

Mark only one circle.

- Yes
- No
- Vaguely

120. Did your loved one know the signs or symptoms of colorectal cancer BEFORE being diagnosed?

Mark only one circle.

- Yes
- No
- Vaguely

Never Too Young - Patient & Caregiver Experiences with Early Onset Colorectal Cancer in Canada Survey 2022

Caregiver: Details of Your Loved One's Colorectal Cancer

121. What year was your loved one diagnosed with colorectal cancer?

122. How old was your loved one when they were diagnosed with colorectal cancer
Mark only one circle.

- Older than 55
- 45-55
- 40-44
- 35-39
- 30-34
- 25-29
- 20-24
- Younger than 20

123. What type of cancer was your loved one diagnosed with (primary tumour)?

- Colon
- Rectal
- I don't know

124. What side was your loved one's primary tumour located on?

Right sided is defined as cancer of the cecum and the ascending colon up to the hepatic flexure. Left-sided colon cancer is defined as cancer of the splenic flexure and cancer in regions distal to the splenic flexure, including the rectum.
(Source: Right-Sided vs Left-Sided Colorectal Cancer – Hematology & Oncology (hematologyandoncology.net))

- Left
- Right
- I don't know

125. What stage colorectal cancer was your loved one diagnosed with?

Stage 0 - the tumour is usually small and has not spread outside the inner lining,

Stage l – the tumour has invaded the layers of the colon or rectum but has not grown outside the walls of the colon or into the surrounding tissue.

Stages II – the tumour has grown through the walls of the colon or rectum into nearby tissue but has not affected lymph nodes.

Stage III - the tumor is larger and has spread outside the colon or rectum to nearby tissues but not to distant organs,

Stage IV (metastatic) - the cancer has spread through blood or lymphatic system to a distant site in the body most commonly the liver, lungs, or peritoneum

- ○ 0
- ○ I
- ○ II
- ○ III
- ○ IV (metastatic)
- ○ I don't know

126. If applicable, select the locations of your loved one's metastases:
Check all that apply.

- ☐ Lung
- ☐ Liver
- ☐ Peritoneum
- ☐ Lymph nodes
- ☐ Bone
- ☐ Spine
- ☐ Brain
- ☐ Prefer not to answer
- ☐ N/A
- ☐ Other (please specify)

127. If applicable, how long has your loved one been NED (No Evidence of Disease)
Mark only one circle .

- ◯ Less than 1 year
- ◯ 1-2 years
- ◯ 2-3 years
- ◯ 3-4 years
- ◯ 4-5 years
- ◯ 5-6 years
- ◯ 6-7 years
- ◯ 7-8 years
- ◯ 8-9 years
- ◯ 9-10 years
- ◯ Longer than 10 years
- ◯ N/A

128. If your loved one has had a recurrence, how many recurrences have they had?

- ◯ 1
- ◯ 2
- ◯ 3
- ◯ 4
- ◯ 5
- ◯ More than 5
- ◯ N/A

Caregiver: Risk Factors

129. Did your loved one have a family history of colorectal cancer in first or second-degree relatives?

A first-degree relative is a close blood relative (full siblings, parents and children).

A second-degree relative is a more distant blood relative (grandparents, nieces, nephews, aunts, uncles, half-siblings).
Mark only one circle

- ◯ Yes
- ◯ No
- ◯ I don't know

130. Prior to diagnosis, did your loved one have a personal history of the following?
Check all that apply.

- ☐ Colitis
- ☐ Crohn's
- ☐ IBD/IBS
- ☐ Adenomatous polyps
- ☐ GI cancers
- ☐ Endometrial cancer
- ☐ Ovarian cancer
- ☐ Breast cancer
- ☐ Prostate cancer
- ☐ No history
- ☐ I don't know

131. Did your loved one have a family history of the following?
Check all that apply.

- [ ] Colitis
- [ ] Crohn's
- [ ] IBD/IBS
- [ ] Adenomatous polyps
- [ ] GI cancers
- [ ] Endometrial cancer
- [ ] Ovarian cancer
- [ ] Breast cancer
- [ ] Prostate cancer
- [ ] No history
- [ ] I don't know

132. Was your loved one a smoker at the time of diagnosis, if yes, about how many packs of cigarettes would they smoke per week?
Mark only one circle.

- ◯ No
- ◯ Yes
- ◯ If yes, then how many packs per week?

133. Was your loved one a smoker in the past, if yes, about how many packs of cigarettes would they smoke per week?
Mark only one circle.

- ◯ No
- ◯ Yes
- ◯ If yes, then how many years of smoking?

134. Did your loved one regularly consume alcohol prior to their diagnosis? If yes, about how many drinks per week would they consume on average?
Mark only one circle.

- ◯ No
- ◯ Yes
- ◯ If Yes (how many drinks per week)

135. Did your loved one regularly consume sugar-sweetened beverages prior to their diagnosis (e.g. soda)?
Mark only one circle.

- Frequently
- Occasionally
- Rare to never

136. How would you rate your loved one's level of physical activity prior to the diagnosis?

Sedentary = less than 30 minutes of moderate/vigorous activity per week,

Lightly active = 30 - 60 minutes of moderate/vigorous activity per week,

Moderately active = 60 - 150 minutes of moderate/vigorous activity per week,

Highly active = 150 + minutes of moderate/vigorous activity per week.

Moderate-intensity aerobic activity makes you breathe harder and your heartbeat faster. You should be able to talk, but not sing. Vigorous-intensity aerobic activity makes your heart rate increase quite a bit and you won't be able to say more than a few words without needing to catch your breath. (Source: Physical Activity Tips for Adults (18-64 years) - Canada.ca)

- Sedentary
- Lightly active
- Moderately active
- Highly active

137. Did your loved one follow any of the following diets prior to diagnosis?
Check all that apply.

- [ ] No specific diet
- [ ] Dairy-free
- [ ] Gluten-free
- [ ] Vegetarian
- [ ] Vegan
- [ ] Mediterranean diet
- [ ] Keto Pescatarian Prefer
- [ ] not to answer Other
- [ ] (please specify)

Caregiver: Symptoms

138. Which of the following symptoms(s) did your loved one experience first? Check all that apply.

- [ ] No symptoms
- [ ] Diarrhea
- [ ] Constipation
- [ ] Weight loss
- [ ] Blood in stool
- [ ] Rectal bleeding
- [ ] Weakness and/or fatigue
- [ ] Persistent cramps
- [ ] Bowel never feels empty
- [ ] Bloating and/or gas
- [ ] Nausea and/or vomiting Pain Other
- [ ] (please specify)

139. Which of the following symptoms appeared subsequently? Check all that apply.

- [ ] No symptoms
- [ ] Diarrhea
- [ ] Constipation
- [ ] Weight loss
- [ ] Blood in stool
- [ ] Rectal bleeding
- [ ] Weakness and/or fatigue
- [ ] Persistent cramps
- [ ] Bowel never feels empty
- [ ] Bloating and/or gas
- [ ] Nausea and/or vomiting Pain Other
- [ ] (please specify)

140. At what age (years) did your loved one first notice symptoms?

141. Which doctors did your loved one see once they noticed symptoms? Please check all that apply.

- [ ] Primary care provider
- [ ] Pediatrician
- [ ] Gastroenterologist
- [ ] Emergency Room Doctor
- [ ] OBGYN
- [ ] Urologist
- [ ] Urgent care
- [ ] Other (please specify)

142. How many different doctors did your loved one see before they were diagnosed with colorectal cancer?
Mark only one circle.

- ○ 1
- ○ 2
- ○ 3
- ○ 4
- ○ 5
- ○ More than 5 doctors

143. How long did it take for your loved one to be diagnosed with colorectal cancer after they first sought medical attention for their symptoms?

○ Less than 1 month ○

1-3 months

○ 3-6 months

○ Longer than 6 months I

○ don't know

144. Did your loved one feel that their doctors dismissed the signs and symptoms of colorectal cancer due to their age?
Mark only one circle.

- ◯ Yes
- ◯ No
- ◯ I don't know
- ◯ Yes, but for a reason other than age.

145. Do you believe that your loved one was MISTAKENLY diagnosed with any of these conditions prior to their diagnosis of colorectal cancer?
Check all that apply

- ☐ Hemorrhoids Symptoms
- ☐ ofchildbirth
- ☐ Appendicitis
- ☐ Gynecological issues IBS
- ☐ IBD
- ☐ Mental health issues
- ☐ Crohn's/Colitis
- ☐ Other (please specify)

- ☐ None of the above

146. Do you believe that your loved one was CORRECTLY diagnosed with any of these conditions prior to your diagnosis of colorectal cancer?
Check all that apply.

- ☐ Hemorrhoids Symptoms
- ☐ ofchildbirth
- ☐ Appendicitis
- ☐ Gynecological issues IBS
- ☐ IBD
- ☐ Mental health issues
- ☐ Crohn's/Colitis
- ☐ Other (please specify)

- ☐ None of the above

Caregiver: Diagnosis

147. Which doctor did your loved one see after diagnosis?
Check all that apply.

- [ ] Family/primary care provider
- [ ] Gastroenterologist Oncologist
- [ ] Pediatrician
- [ ] Surgeon
- [ ] Other (please specify)

148. Did your loved one have genetic testing before or after being diagnosed with colorectal cancer?
Mark only one circle.

- ◯ Yes, before diagnosis
- ◯ Yes, after diagnosis
- ◯ No
- ◯ I don't know

149. If yes, did your loved one test positive for genetic variant, or syndrome such as Lynch Syndrome?
Mark only one circle

- ◯ Yes
- ◯ No
- ◯ If Yes, Signify Variant / Syndrome if known

150. Did your loved one have surgery before consulting with an oncologist?
Mark only one circle.

- ◯ Yes
- ◯ No

151. Did your loved one seek a second opinion with a doctor about treatment options?
Mark only one circle

- Yes
- No

152. Did you and your loved one feel fully informed about their treatment plan and side effects when it was explained by their doctor?
Mark only one circle

- Yes
- No
- I don't know

Caregiver: Biomarkers & Treatment

Biomarkers, short for biological markers, are biological molecules found in blood, other body fluids or tissues that may provide information about a tumour. Results of a biomarker test can help create a more personalized approach to cancer care that will allow you to make more informed decisions.

153. Was biomarker testing a part of your loved one's early age onset colorectal cancer treatment care path?

Select "yes" if biomarker testing was introduced to you, even if you did not go ahead with testing. If biomarker testing was not a part of your treatment care path, select "no" and you will be redirected to the next section of the survey

- ◯ Yes
- ◯ No (Skip to question 158)
- ◯ I don't know (Skip to question 158)

154. Did your loved one's oncologist or any other member of their medical team explain biomarker testing (or tumour profiling) before treatment started?
Mark only one circle

- ◯ Yes
- ◯ No
- ◯ I don't know

155. Did your loved one have their tumour(s) tested for biomarkers?
Mark only one circle

- ◯ Yes
- ◯ No
- ◯ I don't know

156. If yes, do you know the results of your loved one's biomarker testing? Check all that apply.

Check all the apply.

- [ ] MSI-H or deficient MMR
- [ ] MSS or proficient MMR
- [ ] K-RAS mutant
- [ ] K-RAS wild type
- [ ] N-RAS mutant N-
- [ ] RAS wild type
- [ ] BRAF mutant
- [ ] HER2
- [ ] NTRK
- [ ] PIK3CA
- [ ] BRCA1
- [ ] N/A
- [ ] I don't know
- [ ] Other (please specify)

157. If your loved one had their biomarkers tested, did their doctor use the results to help select a treatment that is best for them?

Mark only one circle.

- ○ Yes
- ○ No
- ○ I don't know
- ○ N/A
- ○ Other (please specify)

Caregiver: Clinical Trials

158. Were clinical trials a part of your loved one's early age onset colorectal cancer care path?

Select "yes" if clinical trials were introduced as an option, even if your loved one did not participate in a clinical trial. If clinical trials were not a part of their care path, select "no" and you will be redirected to the next section of the survey
Mark only one circle

- Yes
- No (Skip to Question 166)
- I don't know (Skip to Question 166)

159. Did your loved one's doctor mention clinical trials at diagnosis?
Mark only one circle

- Yes
- No
- I don't remember

160. If you or your loved one heard about clinical trials from another source, please specify below:

161. Did you and/or your loved one feel sufficiently informed about clinical trials?
Mark only one circle

- Yes
- No
- I don't know

162. How important do you believe clinical trials are as treatment options for young-onset colorectal cancer patients?
Mark only one circle.

- Very important
- Somewhat important
- Not important
- I don't know

163. Please indicate how strongly you agree or disagree with the following:
Mark only one circle per row:

	N/A	Strongly Disagree	Disagree	Neutral	Agree	Strongly Agree
Searching for a clinical trial was easy for my loved one	◯	◯	◯	◯	◯	◯
Searching for a clinical trial was overwhelming for my loved one	◯	◯	◯	◯	◯	◯
It was easy for myself and for my loved one to understand the patient information of the clinical trial	◯	◯	◯	◯	◯	◯
My loved one's medical team strongly encouraged me to participate in a clinical trial	◯	◯	◯	◯	◯	◯
My loved one's medical team was not interested in clinical trials	◯	◯	◯	◯	◯	◯

164. Did your loved one participate in a clinical trial?
Mark only one circle.

◯ Yes

◯ No

◯ I don't know

◯ Prefer not to answer

165. If you would like, please share your loved one's experience with clinical trials from your perspective.

Caregiver: Treatment

166. Was your loved one a candidate for surgery at diagnosis?
Mark only one circle.

- Yes
- No
- Prefer not to answer
- I don't know

167. If yes, what type of surgery?

- Right hemicolectomy
- Sigmoid colectomy
- Left hemicolectomy
- Lower anterior resection
- Other (please specify)

168. Did your loved one have radiation?
Mark only one circle.

- Yes
- No
- Prefer not to answer
- I don't know

169. Following your loved one's surgery, did they have a colostomy or ileostomy?
Mark only one circle.

- Yes
- No
- Prefer not to answer
- N/A

Caregiver: Challenges

170. Did any of your loved one's providers discuss sexual side effects due to radiation and surgery before treatment began? Check all that apply.

Check all that apply

- [ ] Painful sex
- [ ] Decrease in sex drive
- [ ] Loss of sexual function
- [ ] Infertility
- [ ] I don't remember
- [ ] Providers did not discuss any of the above

171. Please rate the level of sexual function issues that your loved one experienced after treatment and surgery

Check all that apply

- [ ] Painful sex
- [ ] Decrease in sex drive
- [ ] Loss of sexual function
- [ ] Infertility
- [ ] I don't remember
- [ ] Providers did not discuss any of the above

172. Did your loved one receive any guidance from medical staff regarding sexual health? Check all that apply.

Check all that apply

- [ ] No Guidance Doctors
- [ ] mentioned it
- [ ] Specialist referral
- [ ] Materials Provided
- [ ] I don't know

173. Did a medical professional talk to you and/or your loved one about fertility preservation at any point during diagnosis or treatment?
Mark only one circle.

- Yes
- No
- Prefer not to answer
- N/A

174. Did your loved one have reproductive health issues before treatment?
Mark only one circle.

- Yes
- No
- I don't know
- N/A

175. Did treatment leave your loved one infertile or sterile?
Mark only one circle.

- Yes
- No
- Prefer not to answer
- N/A

176. If you like, please share you and your loved one's experience with changes in sexual or reproductive health due to treatment.

Caregiver: Mental Health

177. Please rate your loved one's experiences while undergoing treatment below.

Please mark only one circle per row -

	Never	Sometimes	Often	Always	Prefer not to answer
My loved one feels / felt emotionally exhausted	○	○	○	○	○
My loved one is withdrawn / withdrew from other people	○	○	○	○	○
My loved one feels / felt like they needs / needed help for their depression	○	○	○	○	○
My loved one feels satisfied with how they are coping with their illness	○	○	○	○	○
My loved one is able to enjoy life	○	○	○	○	○
My loved one is afraid that their cancer may recur	○	○	○	○	○
Thoughts of recurrence interfere with my loved one's daily activities	○	○	○	○	○
I am / was concerned about my loved one's mental health	○	○	○	○	○
My loved one feels / felt sudden feelings of panic and anxiety	○	○	○	○	○
How often did your loved one have to limit social activities due to fatigue?	○	○	○	○	○
How often did your loved one fatigue make it difficult to make decisions?	○	○	○	○	○
How often was your loved one too tired to do everyday	○	○	○	○	○

activities?

How much was your loved one worry about pain?	◯	◯	◯	◯	◯
How much does / did pain interfere with your loved one's family and day - to - day life?	◯	◯	◯	◯	◯

178. Please rate your own experiences below.
Mark only one circle per row.

	Never	Sometimes	Often	Always	Prefer not to answer
I feel / felt emotionally exhausted					
I have withdrawn / withdrew from other people					
I feel / felt like I need / needed help for my depression					
I feel satisfied with how I am coping with my illness					
I am able to enjoy life					
I am afraid that my loved one's cancer may recur					
Thoughts of recurrence interfere with my daily activities					
I am / was concerned about my mental health					
I feel / felt sudden feelings of panic and anxiety					
How often did you have to limit social activities due to fatigue?					
How often did your fatigue make it difficult to make decisions?					
How often were you too tired to do everyday activities?					
How much do / did you worry about loved one's pain?					
How much does / did your loved one' spain interfere with your family and day - to - day life?					

Caregiver: Financial Experience

179. What was your loved one's employment status at the time of diagnosis?
Mark only one circle.

- Employed
- Unemployed
- Retired
- Prefer not to answer
- Other (please specify)

180. What was your employment status at the time of your loved one's diagnosis?

- Employed
- Unemployed
- Retired
- Prefer not to answer
- Other (please specify)

181. Did you and/or your loved one take a leave of absence, quit a job or leave school because of their diagnosis?
Mark only one circle.

- Yes, both of us
- Yes, only my loved one
- Yes, only myself
- No

182. Was the Canada Employment Sickness Benefit sufficient for your loved one?
Mark only one circle.

- Yes
- No
- Prefer not to answer
- N/A

183. Did your loved one have private employment insurance coverage?
Mark only one circle.

- ◯ Yes
- ◯ No
- ◯ N/A

184. Did your employer(s)/school(s) help accommodate your loved one's treatment schedule?
Mark only one circle per row:

- ◯ Yes
- ◯ No
- ◯ Prefer not to answer
- ◯ N/A

185. Please rate your experiences below regarding your own personal financial situation.
Please mark only one circle per row -

	Strongly Disagree	Disagree	Neutral	Agree	Strongly Agree	Prefer not to answer
I feel financially stressed	◯	◯	◯	◯	◯	◯
I worry about the loss of my family's financial stability because of the cost of cancer care for my loved one	◯	◯	◯	◯	◯	◯
I worry about the financial problems I will have in the future due to my loved one's illness / treatment	◯	◯	◯	◯	◯	◯
My loved one's insurance(s) offer(ed) adequate coverage for their treatment needs	◯	◯	◯	◯	◯	◯
I feel in control of my financial situation	◯	◯	◯	◯	◯	◯
I am optimistic about my financial future	◯	◯	◯	◯	◯	◯

Caregiver: Impact of Covid – 19

186. Was your loved one diagnosed/ did they receive treatment after the initial outbreak of the Covid-19 pandemic?

- ◯ Yes
- ◯ No (skip to question 198)

187. What precautions did your loved one take to reduce your risk when attending medical appointments?
Check all that apply.

- ☐ Surgical Gown
- ☐ Face Mask
- ☐ Gloves
- ☐ Glasses/ Googles
- ☐ Hand Washing
- ☐ Other (please specify)

188. Due to Covid-19, was there a delay in your loved one's screening for colorectal cancer? Mark only one circle.

- ◯ No
- ◯ I don't know
- ◯ N/A
- ◯ Yes (if yes for how long?)

189. Due to Covid-19, was there a delay in your loved one’s cancer imaging?
Mark only one circle.

- ◯ No
- ◯ I don't know
- ◯ N/A
- ◯ Yes (if yes for how long?)

190. Due to Covid-19, was there a delay with your loved one’s surgery?
Mark only one circle.

- No
- I don't know
- N/A
- Yes (if yes for how long?)

191. Due to Covid-19, was there a delay with your loved one’s radiation therapy?
Mark only one circle.

- No
- I don't know
- N/A
- Yes (if yes for how long?)

192. Due to Covid-19, was there a delay with your loved one’s cancer treatment (chemotherapy, immunotherapy)?
Mark only one circle.

- Yes
- No
- I don't know
- N/A

193. Due to Covid-19, did your loved one have appointments virtually?
Mark only one circle.

- Yes
- No
- N/A

194. If yes, would your loved one have preferred to have had their appointment(s) in person?
Mark only one circle.

- Yes
- No
- I don't know
- N/A

195. Due to Covid - 19, which of these factors has made an impact on your loved one’s access to cancer care?
Check all that apply.

- [ ] No changes in care
- [ ] Care delayed
- [ ] Location of care switched
- [ ] Treatment switched Other
- [ ] (please specify)

196. Due to Covid-19, was there a delay in your loved one’s clinical trial?
Mark only one circle.

- ( ) No
- ( ) I don't know
- ( ) N/A
- ( ) Yes (if so for how long?)

197. Has Covid - 19 affected your loved one’s social determinants of health?
Check all that apply.

- [ ] Access to transportation
- [ ] Access to healthcare clinics / hospitals
- [ ] Difficulties getting childcare
- [ ] Job Loss
- [ ] Access to adequate food
- [ ] Access to pharmacies / acquiring medications
- [ ] Other (please specify)

Caregiver: Summary

198. What is the biggest challenge you've faced as a caregiver for someone diagnosed under 50?

199. The caregiver survey questions have now ended. Please select "finished" to proceed to the conclusion section.

Finished (skip to question 200).

Never Too Young - Patient & Caregiver Experiences with Early Onset Colorectal Cancer in Canada Survey 2022

Conclusion -

200. Please select all that apply.

- [ ] I would be interested in getting more information about an early age onset colorectal cancer support group or caregiver support group
- [ ] I wish to receive Colorectal Cancer Canada's newsletter
- [ ] I may be interested in participating in future surveys regarding early age onset colorectal cancer
- [ ] I would like to share my early age onset CRC story on Colorectal Cancer Canada's website (https://www.colorectalcancercanada.com/stories/)

201. Would you be interested in viewing early age onset colorectal cancer webinars?

If yes, please select the topics that you would be interested in. If you would be interested in a topic area not listed here, please specify in the 'other' category.

- [ ] I would not be interested in viewing EAO CRC webinars
- [ ] Expert Q&A webinars
- [ ] Emerging research and treatment options
- [ ] Mental wellness, relationships and sexuality webinars
- [ ] Caregiver support webinars
- [ ] Nutrition and physical activity webinars Other
- [ ] (please specify)

202. If you selected any of the options above, please leave your email address here. Please note, you will only be contacted regarding the option(s) that you selected.

203. We would like to thank you for taking the time to complete this survey.

We will be randomly selecting ten participants to receive $50 Lululemon e-gift cards. If interested, please leave your email address below to enter the draw.

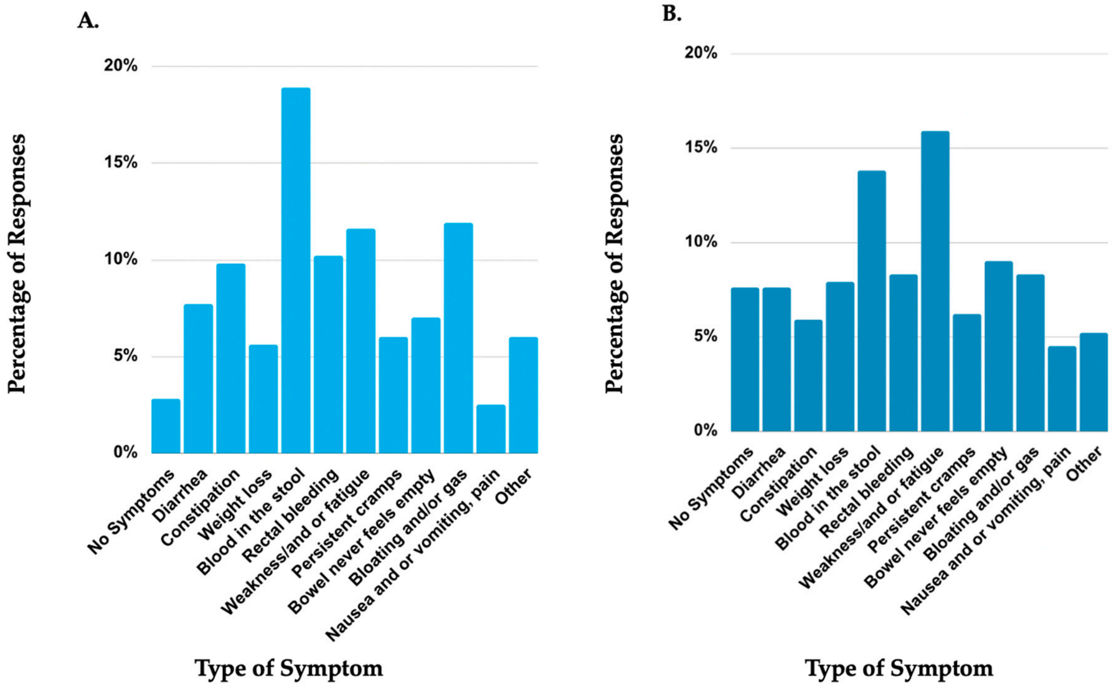

Figure S1: Symptoms experienced by early age onset colorectal cancer (EOCRC) patients. (**A**) First symptoms to appear (N =123). (**B**) Subsequent symptoms to appear following symptoms onset (N = 115).

Table S1: Respondent demographics for the Never Too Young survey

Variable	Total N	%
Connection to Colorectal Cancer		
Patient	69	53.9
Survivor	39	30.5
Caregiver	20	15.6
Current Age		
20-29	2	1.9
30-39	18	16.7
40-49	48	44.4
50-59	31	28.7
60+	9	8.3

**Race/Ethnicity**		
Arab	2	1.6
Ashkenazi Jewish	3	2.4
Black	1	0.8
Chinese	1	0.8
Japanese	1	0.8
Korean	1	0.8
Latin American	1	0.8
First Nations	2	1.6
White	111	87.4
Prefer not to answer	1	0.8
Other	3	2.4
**Gender**		
Female	80	63.5
Male	45	35.7
Non-Binary	1	0.8
**Marital Status at Diagnosis**		
Married / Common Law	94	73.4
Single	22	17.2
Divorced	9	7.0
Other	3	2.3
**Children at Diagnosis**		
Yes	87	69.0
No	39	31.0
**Education**		
High school degree or equivalent	21	16.5
Cégep / college diploma/trade college	36	28.3
Bachelor's degree	49	38.6
Master's degree	17	13.4
Doctorate	2	1.6
Other	2	1.6
**Community Type**		
Urban	58	46.0
Suburban	39	31.0
Rural	29	23.0

**Province of Residence**		
Alberta	13	10.2
British Columbia	12	9.4
Manitoba	3	2.4
New Brunswick	4	3.1
Newfoundland & Labrador	4	3.1
Nova Scotia	5	3.9
Ontario	65	51.2
Prince Edward Island	1	0.8
Quebec	14	11.0
Saskatchewan	6	4.7

Table S2: The awareness of early-age onset colorectal cancer (EOCRC), health history and risk factors for colorectal cancer (CRC)

Variable	Total N	%
Early Age Onset Colorectal Cancer Awareness		
**Awareness that colorectal cancer can occur in those under 50**		
Yes	33	26.4
No	52	41.6
Vaguely	40	32.0
**Know the signs and symptoms of colorectal cancer before diagnosis**		
Yes	30	24.2
No	56	45.2
Vaguely	38	30.6
**Discuss family health history including colorectal cancer risk factors before diagnosis**		
Family and or friends	2	1.9
Doctor	10	9.3
Family and/or friends and doctor	17	15.9
Did not discuss	78	72.9

**Health History**		
Family history of colorectal cancer		
Yes	52	41.9
No	64	51.6
I don't know	8	6.5
Family history of disease		
Colitis	13	7.6
Crohn's	9	5.2
IBD/IBS	13	7.6
Adenomatous polyps	6	3.5
GI cancers	14	8.1
Endometrial cancer	4	2.3
Ovarian cancer	11	6.4
Breast cancer	27	15.7
Prostate cancer	16	9.3
No history	48	27.9
I don't know	11	6.4
Personal history of disease		
Colitis	5	3.8
IBD/IBS	11	8.4
Adenomatous polyps	3	2.3
Breast cancer	3	2.3
No history	109	83.2
BMI		
<18.5	2	2.0
18.5-24.9	35	34.7
25-29.9	40	39.6
30+	24	23.8
**Behavioural Risk Factors**		
Consumption of sugar-sweetened beverages		
Frequently	30	24.2
Occasionally	43	34.7
Rarely	51	41.1
Physical activity		
Sedentary	5	4.0

Lightly active	45	36.3
Moderately active	42	33.9
Highly active	32	25.8
Smoker at the time of diagnosis		
Yes	13	10.3
No	110	89.7
Smoker in the past		
Yes	34	32.4
No	71	67.6
If yes, average number of years		
<5	5	16.1
5-10	12	38.7
11-14	4	12.9
15-20	4	12.9
20+	6	19.4
Consume alcoholic beverages		
Yes	67	54.5
No	56	45.5
If yes, how many drinks per week		
0-5	37	58.7
6-15	20	31.7
15+	6	9.5
Diet		
No specific diet	101	75.4
Dairy-free	4	3.0
Gluten-free	5	3.7
Vegetarian	5	3.7
Vegan	2	1.5
Mediterranean diet	3	2.2
Keto Pescatarian	6	4.5
Other (please specify)	8	6.0
Vegetable consumption		
0 Servings	2	1.9
1 Serving	22	21.2
2 Servings	25	24.0
3 Servings	22	21.2
4 Servings	14	13.5
5 Servings	8	7.7

6 Servings	5	4.8
7 Servings	3	2.9
8 Servings	2	1.9
More than 8 Servings	1	1.0
**Fruit consumption**		
0 Servings	5	4.8
1 Serving	28	26.9
2 Servings	32	30.8
3 Servings	21	20.2
4 Servings	14	13.5
5 Servings	3	2.9
6 servings	0	0.0
7 Servings	1	1.0
8 servings	0	0.0

Table S3: Characteristics of colorectal cancer (CRC) diagnosis among respondents

Variable	Total N	%
**Type of Cancer**		
Colon	71	57.3
Rectal	50	40.3
I don’t know	3	2.4
**Location of cancer**		
Left	60	48.8
Right	29	23.6
I don't know	34	27.6
**Stage of Cancer at Diagnosis**		
0	4	3.2
I	15	12.0
II	11	8.8
III	60	48.0
IV (metastatic)	31	24.8
I don't know	4	3.2
**Location of Metastases**		
Lung	21	13.7
Liver	31	20.3
Peritoneum	8	5.2

Lymph nodes	36	23.5
Bone	3	2.0
Spine	2	1.3
Brain	3	2.0
N/A	49	32.0

Table S4: Treatment experience of early-age onset colorectal cancer (EOCRC) patients

Variable	Total N	%
Surgery before consulting with an oncologist		
Yes	55	47.4
No	61	52.6
Understood and felt fully informed on treatment plan		
Yes	91	75.2
No	24	19.8
I don't know	6	5.0
Sought a second opinion		
Yes	19	15.7
No	102	84.3
Candidate for surgery at initial diagnosis		
Yes	96	82.8
No	19	16.4
Prefer not to answer	1	0.9
Type of surgery		
Right hemicolectomy	12	13.6
Sigmoid colectomy	21	23.9
Left hemicolectomy	9	10.2
Lower anterior resection	29	33.0
Other	17	19.3
Did you have radiation?		
Yes	53	46.1

No	62	53.9
**Had a colostomy or ileostomy**		
Yes	42	36.8
No	64	56.1
N/A	8	7.0

Table S5: Mental health experience of early-age onset colorectal cancer (EOCRC) patients

Variable	Total N	%
**I feel/felt emotionally exhausted**		
Never	0	0.0
Sometimes	25	22.5
Often	46	41.4
Always	40	36.0
Prefer not to answer	0	0.0
**I have withdrawn/withdrew from other people**		
Never	11	9.9
Sometimes	47	42.3
Often	40	36.0
Always	13	11.7
Prefer not to answer	0	0.0
**I feel/felt like I needed help for my depression**		
Never	32	29.1
Sometimes	37	33.6
Often	26	23.6
Always	15	13.6
Prefer not to answer	0	0.0

I feel satisfied with how I am coping with my illness		
Never	8	7.3
Sometimes	47	42.7
Often	39	35.5
Always	16	14.5
Prefer not to answer	0	0.0
I am able to enjoy life		
Never	5	4.6
Sometimes	40	36.7
Often	44	40.4
Always	20	18.3
Prefer not to answer	0	0.0
I am afraid that my cancer may recur		
Never	4	3.7
Sometimes	24	22.0
Often	16	14.7
Always	62	56.9
Prefer not to answer	3	2.8
Thoughts of recurrence interferes with daily activities		
Never	36	32.7
Sometimes	42	38.2
Often	19	17.3
Always	11	10.0
Prefer not to say	2	1.8
I am/was concerned about my mental health		
Never	12	12.6
Sometimes	46	48.4
Often	23	24.2
Always	14	14.7
Prefer not to say	0	0.0

**I feel/felt sudden feelings of panic and anxiety**		
Never	19	17.1
Sometimes	48	43.2
Often	30	27.0
Always	14	12.6
Prefer not to say	0	0.0
**Had to limit social activities due to fatigue**		
Never	5	4.5
Sometimes	47	42.0
Often	44	39.3
Always	16	14.3
Prefer not to answer	0	0.0
**How often did fatigue make it difficult to make decisions?**		
Never	17	15.3
Sometimes	54	48.6
Often	28	25.2
Always	12	10.8
Prefer not to answer	0	0.0
**How often were you too tired to do everyday activities?**		
Never	2	1.8
Sometimes	52	46.8
Often	45	40.5
Always	12	10.8
Prefer not to answer	0	0.0
**How often do you/ did you worry about pain?**		
Never	26	23.6
Sometimes	45	40.9
Often	24	21.8
Always	15	13.6
Prefer not to answer	0	0.0

**How much does/did pain interfere with your family and day-to-day life?**		
Never	23	21.3
Sometimes	48	44.4
Often	26	24.1
Always	11	10.2
Prefer not to answer	0	0.0

Table S6: Mental health experience of caregivers

Variable	Total N	%
**I am / was concerned about my loved one's mental health**		
Never	2	12.5
Sometimes	6	37.5
Often	2	12.5
Always	6	37.5
Prefer not to answer	0	0.0
**I feel / felt emotionally exhausted**		
Never	0	0.0
Sometimes	1	6.3
Often	10	62.5
Always	5	31.3
Prefer not to answer	0	0.0
**I have withdrawn / withdrew from other people**		
Never	0	0.0
Sometimes	5	31.3
Often	9	56.3
Always	2	12.5
Prefer not to answer	0	0.0

I feel / felt like I need / needed help for my depression		
Never	6	37.5
Sometimes	5	31.3
Often	2	12.5
Always	3	18.8
Prefer not to answer	0	0.0
I feel satisfied with how I am coping the illness		
Never	3	20.0
Sometimes	9	60.0
Often	1	6.7
Always	1	6.7
Prefer not to answer	1	6.7
I am able to enjoy life		
Never	2	12.5
Sometimes	9	56.3
Often	4	25.0
Always	1	6.3
Prefer not to answer	0	0.0
I am afraid that my loved one's cancer may recur		
Never	0	0.0
Sometimes	1	6.7
Often	3	20.0
Always	10	66.7
Prefer not to answer	1	6.7
Thoughts of recurrence interfere with my daily activities		
Never	2	13.3
Sometimes	6	40.0
Often	3	20.0
Always	3	20.0
Prefer not to answer	1	6.7

I am / was concerned about my mental health		
Never	2	12.5
Sometimes	7	43.8
Often	3	18.8
Always	4	25.0
Prefer not to answer	0	0.0
I feel / felt sudden feelings of panic and anxiety		
Never	2	12.5
Sometimes	4	25.0
Often	7	43.8
Always	2	12.5
Prefer not to answer	1	6.3
How often did you have to limit social activities due to fatigue?		
Never	2	12.5
Sometimes	7	43.8
Often	5	31.3
Always	1	6.3
Prefer not to answer	1	6.3
How often did your fatigue make it difficult to make decisions?		
Never	5	31.3
Sometimes	6	37.5
Often	4	25.0
Always	0	0.0
Prefer not to answer	1	6.3
How often were you too tired to do everyday activities?		
Never	6	37.5
Sometimes	7	43.8
Often	3	18.8
Always	0	0.0

Prefer not to answer	0	0.0
**How much do / did you worry about loved one's pain?**		
Never	1	6.3
Sometimes	2	12.5
Often	5	31.3
Always	8	50.0
Prefer not to answer	0	0.0
**How much does / did your loved one' s pain interfere with your family and day - to - day life?**		
Never	4	25.0
Sometimes	7	43.8
Often	4	25.0
Always	1	6.3
Prefer not to answer	0	0.0

Table S7: Financial health among patients and caregivers

Variable	Total N	%
**Patient employment status at diagnosis**		
Employed	96	85.0
Unemployed	6	5.3
Retired	2	1.8
Prefer not to answer	1	0.9
Other	8	7.1
**Caregiver employment status at diagnosis**		
Employed	11	73.3
Unemployed	1	6.7
Retired	0	0.0
Prefer not to answer	0	0.0
Other	3	20.0

**Patient had to take a leave of absence, quit job, or left school because of diagnosis**		
Yes	72	81.6
No	17	18.4
**As a caregiver did you or your loved one take a leave of absence, quit a job, or leave school due to diagnosis**		
Yes, both of us	4	26.7
Yes, only my loved one	9	60.0
Yes, only myself	0	0.0
No	2	13.3
**Was the Canada Employment Sickness Benefit sufficient?**		
Yes	6	5.4
No	37	33.0
Prefer not to answer	1	0.9
N/A	68	60.7
**Did you have private employment insurance coverage?**		
Yes	66	59.5
No	35	31.5
N/A	10	9.0
**Employer/school helped accommodate treatment schedule**		
Yes	64	57.1
No	14	12.5
N/A	34	30.4

I feel financially Stressed		
Strongly Disagree	18	16.1
Disagree	18	16.1
Neutral	21	18.8
Agree	27	24.1
Strongly Agree	27	24.1
Prefer not to say	1	0.9
I worry about the loss of my family's financial stability because of the cost of cancer care		
Strongly Disagree	25	22.3
Disagree	21	18.8
Neutral	22	19.6
Agree	23	20.5
Strongly Agree	20	17.9
Prefer not to say	1	0.9
I worry about the financial problems I will have in the future due to illness / treatment		
Strongly Disagree	17	15.3
Disagree	12	10.8
Neutral	20	18.0
Agree	36	32.4
Strongly Agree	25	22.5
Prefer not to say	1	0.9
I worry that my public insurance is not adequate enough to cover my cost of cancer care and treatment		
Strongly Disagree	20	21.5
Disagree	15	16.1
Neutral	20	21.5
Agree	19	20.4
Strongly Agree	15	16.1
Prefer not to say	4	4.3

**Feel in control of financial situation**		
Strongly Disagree	9	8.3
Disagree	20	18.3
Neutral	36	33.0
Agree	27	24.8
Strongly Agree	17	15.6
Prefer not to say	0	0.0
**Feel optimistic about financial future**		
Strongly Disagree	12	11.0
Disagree	23	21.1
Neutral	34	31.2
Agree	25	22.9
Strongly Agree	14	12.8
Prefer not to say	1	0.9

Table S8: Clinical trial experience of early-age onset colorectal cancer (EOCRC) patients

Experience With Clinical Trial	Total N	%
**Clinical trials as part of care path**		
Yes	23	19.5
No	86	72.9
I don't know	9	7.6
**Doctor mentioned clinical trials at diagnosis**		
Yes	18	42.9
No	17	40.5
I don't remember	7	16.7
**Felt sufficiently informed about clinical trials**		

Yes	22	57.9
No	16	42.1
**Importance of clinical trials as treatment options for early age onset colorectal cancer patients**		
Very important	30	78.9
Somewhat important	8	21.1
Not important	0	0.0
**Would you consider participating in a clinical trial?**		
Yes	23	74.2
No	8	25.8
**Did you participate in a clinical trial?**		
Yes	16	39.0
No	24	58.5
Prefer not to answer	1	2.4
